# Predicting a Kind of Unusual Multiple-States Dimerization-Modes Transformation in Protein PD-L1 System by Computational Investigation and a Generalized Rate Theory

**DOI:** 10.3389/fchem.2021.783444

**Published:** 2021-11-09

**Authors:** Zhong-Xing Zhou, Hong-Xing Zhang, Qing-Chuan Zheng

**Affiliations:** ^1^ Institute of Theoretical Chemistry, College of Chemistry, Jilin University, Changchun, China; ^2^ Key Laboratory for Molecular Enzymology and Engineering of the Ministry of Education, College of Life Science, Jilin University, Changchun, China

**Keywords:** self-assembly network, molecular dynamics, dimerization-modes stability, self-assembly transformation kinetic rate, PD-L1

## Abstract

The new cancer immunotherapy has been carried out with an almost messianic zeal, but its molecular basis remains unclear due to the complexity of programmed death ligand 1 (PD-L1) dimerization. In this study, a new and integral multiple dimerization-modes transformation process of PD-L1s (with a new PD-L1 dimerization mode and a new transformation path discovered) and the corresponding mechanism are predicted using theoretical and computational methods. The results of the state analysis show that 5 stable binding states exist in system. A generalized inter-state transformation rate (GITR) theory is also proposed in such multiple-states self-assembly system to explore the kinetic characteristics of inter-state transformation. A “drug insertion” path was identified as the dominant path of the PD-L1 dimerization-modes transformation. Above results can provide supports for both the relative drug design and other multiple-states self-assembly system from the theoretical chemistry perspective.

## Introduction

The search for a cure for cancer has been one of the great pursuits of modern science. Cancer immunotherapy has been called the third revolution in cancer therapy (Miller and Sadelain, 2015), bringing hope for a cure for many types of cancer (Mahoney et al., 2015; Hoos, 2016; Hu and Li, 2020). One of the main mechanisms of cancer immunotherapy is to block the binding between the receptor, known as programmed death protein 1 (PD-1), on the surface of immune cells and the ligand, known as programmed death ligand 1 (PD-L1), on the surface of cancer cells to prevent cancer cells immune evasion, so as to promote the recognition and the targeted killing of cancer cells by the body’s immune cells (Ishida et al., 1992; Sharma and Allison, 2015).

The PD-1/PD-L1 target of cancer immunotherapy has already exhibited excellent clinical pharmacological effects. For example, it has been a great success to block the binding of PD-1/PD-L1 with monoclonal antibody in clinical application (Brahmer et al., 2012; Topalian et al., 2012). However, the antibody drugs have some inherent disadvantages such as high price, short half-life, poor penetrability and immunogenicity (Chames et al., 2009). Fortunately, these disadvantages can be overcome by the development of small-molecule PD-1/PD-L1 blocking drugs. It is a pity that the development of small-molecule drugs that block the PD-1/PD-L1 pathway is relatively lagging currently, which is mainly due to the lack of in-depth understanding of the interaction mechanism between the small-molecule drugs and the PD-L1s.

The Bristol-Myers-Squibb (BMS) team has made a breakthrough in developing a class of effective small-molecule PD-1/PD-L1 blocking drugs based on the (2-methyl-3-biphenylyl) methanol scaffold (Chupak et al., 2017; Chupak and Zheng, 2018), and the mechanism of interaction between the drugs and the proteins had not been reported. Holak et al. solved several holo form (involving small-molecule blocking drugs) sandwich PD-L1 dimeric crystal structures (PDB ID: 5J89, 5J8O, 5N2D, 5NIU, 5N2F, 6R3K, 6RPG) using X-ray (Zak et al., 2016; Guzik et al., 2017; Skalniak et al., 2017; Basu et al., 2019). These holo form dimerization structures in the solid phase crystals provide a prerequisite for relevant studies and greatly contribute to the understanding of the action mechanism of such small-molecule PD-1/PD-L1 blockers. More interestingly, to apo form PD-L1, there are several X-ray diffraction crystal structures (PDB ID: 4Z18, 5JDR, 3FN3) and EGS cross-linking molecular weight evidence indicating that PD-L1s may also be apo form dimer in the absence of drugs (Chen et al., 2010; Zhang et al., 2017). Even so, PD-L1s have different binding interfaces and nearly opposite relative orientation between holo form dimerization mode and apo form dimerization mode.

The above results indicate that PD-L1s may have both holo form dimers and apo form dimers in solution. Together, these results provide complete prerequisite information to explore the integral mechanism of drug involving PD-L1 dimerization, for that it is difficult to recruit macromolecule PD-L1s aggregating entirely by small molecules themselves in physical images. However, currently, the potential transformations between the apo form dimerization modes and the holo form dimerization modes remain ambiguous, as none of the existing studies has adequately considered the forming of holo dimers in relation to the existence of the apo dimers.

To clarify the remaining research space, we address the following questions: To begin with, in the aspect of stable states of the system, there are three questions below. Whether the two kinds of dimerization modes (holo form and apo form) solved using X-ray in solid phase can be stable in solution? Are there any other stable binding states in the solution? What are the stability factors for dimerization modes that can exist in solution? Further, in the aspect of inter-state transitions among the stable states, there are also three more questions. What are the possible self-assembly paths that can happen in kinetics? How to determine the dominant path in this multiple-paths self-assembly system? Ultimately, does this class of small-molecule drugs act as inducers or stabilizers in the formation of the holo form dimerization mode? Combined computations of multi-scale simulations with theoretical deduction, the above questions could be solved, which will, in turn, bridge the gaps within the current research.

Moreover, for the kinetic characteristics in the abstract aspect, such as the dominant path of inter-state transformation in this complex self-assembly network, we proposed a generalized inter-state transformation rate (GITR) theory in a different perspective from the numerical calculation. This work provides additional insights into other similar complex multiple-states self-assembly system.

## Materials and Methods

### Starting Structures in Molecular Dynamics Simulations

The initial structure of apo form dimerization structure 1 was obtained from Protein Data Bank (PDB ID: 4Z18). The two chains of 4Z18 were cut off at the peptide bonds between Tyr134 and Asn135, with the relative position and orientation of the two chains kept unchanged. And then, the upper IgV domain (D1) and the lower IgC domain (D2) of PD-L1 from the initial structure 4Z18 were obtained and renamed as 4Z18^U^ and 4Z18^L^ respectively (Chen et al., 2010). Correspondingly, the initial structure of holo form dimerization structure was obtained from Protein Data Bank (PDB ID: 5J89) (Zak et al., 2016). Similarly, the starting structures of IS^L^, IS^R^ and IS^PP^ were obtained by removing A chain, B chain and drug molecule BMS-202 from the structure of 5J89 respectively, keeping the relative position and orientation of the rest parts unchanged. The H++ online server (Gordon et al., 2005) was used to calculate the protonation states of the ionizable residues at pH = 7.0. The partial charges of drug molecule BMS-202 were calculated by AM1-BCC method (Jakalian et al., 2000). The field parameters of BMS-202 were obtained by using Antechamber Suite with the General AMBER Force Field (GAFF) (Wang et al., 2004). Similarly, the field parameters of PD-L1 were obtained by using t-LEaP module of AMBER 16 package with the ff14SB force field (Maier et al., 2015; Case et al., 2016). Moreover, the missing atoms in the proteins were added by t-LEaP module. In order to make the system electrically neutral, $Na^{+}$ or $Cl^{-}$ models were added as counter ions, with their three-dimensional positions were computed using Coulombic potential grid algorithm (Case et al., 2016). Finally, each system was solvated using the TIP3P water model (Jorgensen et al., 1983) in a truncated octahedron box with a 12.0 Å distance around the solute.

### Molecular Dynamics Simulations

The MD simulations (Alder and Wainwright, 1959; Mccammon et al., 1976; Tuckerman and Martyna, 2000) were carried out using the AMBER 16 software package (Case et al., 2016). Firstly, 20,000 steps of energy minimization (10,000 steps of steepest decent followed by 10,000 steps of conjugate gradient) were carried out with protein and drug constrained (500 kcal mol^−1^ Å^−2^). Subsequently, 20,000 steps of energy minimization were repeated without any constrain. Next, each system was heated linearly from 0 to 310 K over a period of 310 ps with 10.0 kcal mol^−1^ Å^−2^ restrain on the solute and then another 200 ps equilibrium simulation in the NVT ensemble was followed at 310 K with 5.0 kcal mol^−1^ Å^−2^ restrain on the solute. Finally, 150 ns MD simulation was performed for each system in the NPT ensemble to produce trajectory. A constant isotropic pressure of the system was maintained at 1 atm using the Berendsen barostat (Berendsen et al., 1984). Meanwhile, the temperature of each system was maintained at 310 K by coupling to a Langevin heatbath (Uberuaga et al., 2004) using a collision frequency of 1 ps^−1^. Short range nonbonded interactions were cut off at 10.0 Å, while the long-range electrostatic interactions were handled using the particle mesh Ewald (PME) method (Darden et al., 1993). The time step of each MD simulation was set to 2 fs using SHAKE algorithm (Ryckaert et al., 1977) to restrict all covalent bonds involving hydrogen atoms.

### Adaptive Steered Molecular Dynamics Simulations

The starting state of ASMD simulations (Ozer et al., 2010; Ozer et al., 2012) was the last frame in the 150 ns MD simulation of the crystal structure (PDB ID:5J89). The adaptive steering displacement vector was defined with the ALA-B121 Beta C atom in the PD-L1s dimer as the starting point and the C12 atom at the end of drug molecule BMS-202 as the terminal point. The length of the adaptive steering displacement vector was measured by VMD (Humphrey et al., 1996). Considering the length of the drug molecule, we pulled the drug along the displacement vector 20 Å with the magnitude of the vector increasing from 17.28 to 37.28 Å. The pulling speed v and the spring constant k were chosen as 2 Å ns^−1^ and 20 kcal mol^−1^ Å^−2^ respectively. The potential of mean force (PMF), representing the free energy change ΔG along the displacement vector or pulling coordinate, would be computed from ASMD trajectories using Jarzynski’s equality (Ozer et al., 2010; Ozer et al., 2012):

$$e^{\left(-\frac{\Delta G}{kT}\right)}=\langle e^{\left(-\frac{W_{s\to t}}{kT}\right)}\rangle$$ (1)

where the $W_{s\to t}$ is the pulling work from the starting point to the terminal point in trajectories. In order to adapt our ASMD algorithm to the geometry of the sinuous channel between two PD-L1 monomers, we divided 20-Å-pulling coordinate into 10 stages to increase the calculation accuracy. At each stage, steering molecular dynamics simulations were run 20 times independently to enhance the sampling, with 500,000 steps for each trajectory and 0.002 ps for each step. Totally, for each system, 200 ns ASMD simulations were needed to pull the drug out of the channel. The ASMD simulations were executed by the AMBER package (Case et al., 2016).

### Root Mean Square Deviation and Root Mean Square Fluctuations

Root mean square deviation measures the change of the conformation with the change of time, while root mean square fluctuations measure the fluctuations of atoms or residues during the simulations. They are defined below:

$$RMSD\left(t\right)=\sqrt{\frac{\sum_{i}^{N}\left[m_{i}\times {\left(r_{i}\left(t\right)-r_{i}^{\prime }\right)}^{2}\right]}{\sum_{i}^{N}m_{i}}}$$ (2)

$$RMSF_{atom}\left(i\right)=\;Atom_{Fluct_{i}}=\sqrt{\frac{\sum_{t=1}^{Frames}{\left(r_{i}\left(t\right)-r_{i}^{\prime }\right)}^{2}}{Frames}}$$ (3)

$$RMSF_{resid}\left(R\right)=Resid_{Fluct_{R}}=\frac{\sum_{i}^{R}Atom_{Fluct_{i}}\times m_{i}}{\sum_{i}^{R}m_{i}}$$ (4)

Here, the $r_{i}\left(t\right)$ is the position vectors of atom i at time t and the $r_{i}^{\prime }$ is the position vectors of atom in reference structure. Specifically, the N, Frames and R represent summing over each atom in the selected parts of the structure, each frame in the trajectories and each atom in the residue respectively. They were computed using the CPPTRAJ module of the AMBER package (Roe and Cheatham, 2013).

### Dynamic Cross-Correlation Matrix

Firstly, all the conformations in the trajectories would be aligned against a reference structure to remove overall global translational and rotational motions by means of a least-square-fitting procedure using all backbone Cα atoms. Covariance matrix elements are denoted as lower-case $c_{ij}$ while dynamical cross-correlation matrix elements are denoted as upper-case $C_{ij}$ . They are computed by Eqs 5, 6 as shown below (Hunenberger et al., 1995):

$$\begin{array}{l} c_{ij}=\langle \left(r_{i}\left(t\right)-\langle r_{i}\left(t\right)\rangle \right)\left(r_{j}\left(t\right)-\langle r_{j}\left(t\right)\rangle \right)\rangle \\ =\langle r_{i}\left(t\right)r_{j}\left(t\right)\rangle -\langle r_{i}\left(t\right)\rangle \langle r_{j}\left(t\right)\rangle \end{array}$$ (5)

$$C_{ij}=\frac{c_{ij}}{c_{ii}^{1/2}c_{jj}^{1/2}}=\frac{\langle r_{i}\left(t\right)r_{j}\left(t\right)\rangle -\langle r_{i}\left(t\right)\rangle \langle r_{j}\left(t\right)\rangle }{{\left[\left(\langle r_{i}^{2}\left(t\right)\rangle -{\langle r_{i}\left(t\right)\rangle }^{2}\right)\left(\langle r_{j}^{2}\left(t\right)\rangle -{\langle r_{j}\left(t\right)\rangle }^{2}\right)\right]}^{1/2}}\;$$ (6)

Here, the angle brackets denote time averages. The $r_{i}\left(t\right)$ and $r_{j}\left(t\right)$ are the position vectors of atom i and atom j at time t respectively. Dynamical cross-correlation matrix element can be understood as the normalized covariance matrix element with the value from −1 to +1. It reflects the correlation of displacements between two atoms along a straight line. The DCCM were computed by the CPPTRAJ module of the AMBER package (Roe and Cheatham, 2013).

### Binding Free Energy Calculation

Binding free energy $G_{bind}$ can be divided into $G_{mmpbsa}$ term (in which only solvation entropy is considered in the aspect of entropy effect, as defined below) and gas phase conformational entropy term TS , which could be calculated by MM-PBSA method and Normal Mode Analysis method (Miller et al., 2012) respectively:

$$G_{bind}=G_{mmpbsa}-TS$$ (7)

$G_{mmpbsa}$ can be divided into the molecular mechanics energy term $E_{MM}$ and the free energy change of solvation effect $\Delta G_{solv}$ :

$$G_{mmpbsa}=E_{MM}+\Delta G_{solv}$$ (8)

Furthermore, the molecular mechanics energy term $E_{MM}$ can be divided into the internal energy of molecule $\;E_{int}$ , the van der Waals energy $E_{vdw}$ and the electrostatic energy $E_{ele}$ :

$$E_{MM}=E_{int}+E_{vdw}+E_{ele}$$ (9)

Moreover, the internal energy of molecule $E_{int}$ can be divided into the bond energy term $E_{bond}$ , the angle energy term $E_{angle}$ , the torsion energy term $E_{torsion}$ :

$$E_{int}=E_{bond}+E_{angle}+E_{torsion}$$ (10)

Correspondingly, the free energy change of solvation effect $\Delta G_{solv}$ can be divided into the free energy change of polar solvation effect $\Delta G_{PB}$ and the free energy change of nonpolar solvation effect $\Delta G_{SA}$ :

$$\Delta G_{solv}=\Delta G_{PB}+\Delta G_{SA}$$ (11)

Note that the free energy change of polar solvation effect $\Delta G_{PB}$ could be obtained by solving Poisson-Boltzmann equation (Fogolari et al., 1999):

$$\nabla \cdot \left[\varepsilon \left(\vec{r}\right)\nabla Ø\left(\vec{r}\right)\right]=-4\pi \rho \left(\vec{r}\right)-4\pi \lambda \left(\vec{r}\right)\underset{i}{\sum }z_{i}c_{i}e^{-\frac{z_{i}Ø\left(\vec{r}\right)}{k_{B}T}}$$ (12)

where $\varepsilon \left(\vec{r}\right)$ is the dielectric constant, $Ø\left(\vec{r}\right)$ is the electrostatic potential, $\rho \left(\vec{r}\right)$ is the solute charge, $\lambda \left(\vec{r}\right)$ is the Stern layer masking function, $z_{i}$ is the charge of ion type i , $c_{i}$ is the bulk number density of ion type i far from the solute, $k_{B}$ is the Boltzmann constant, and T is the temperature.

The free energy change of nonpolar solvation effect $\Delta G_{SA}$ can be calculated from surface tension γ and solvent accessible surface SASA Sitkoff et al., 1994.

$$\Delta G_{SA}=\gamma SASA+\beta$$ (13)

As a result, the change of binding free energy $\Delta G_{bind}$ can be deduced from $\Delta G_{mmpbsa}$ and the change of gas phase conformational entropy TΔS , as follows:

$$\begin{array}{l} \Delta G_{bind}=G_{liq}^{AB}-\left(G_{liq}^{A}+G_{liq}^{B}\right)\\ \;=G_{gas}^{AB}+\Delta G_{solv}^{AB}-\left(G_{gas}^{A}+\Delta G_{solv}^{A}+G_{gas}^{B}+\Delta G_{solv}^{B}\right)\\ =\left(G_{gas}^{AB}-G_{gas}^{A}-G_{gas}^{B}\right)+\Delta G_{solv}^{AB}-\Delta G_{solv}^{A}-\Delta G_{solv}^{B}\\ =\Delta G_{gas}+\left(\Delta G_{PB}^{AB}+\Delta G_{SA}^{AB}\right)-\left(\Delta G_{PB}^{A}+\Delta G_{SA}^{A}\right)-\left(\Delta G_{PB}^{B}+\Delta G_{SA}^{B}\right)\\ \;=\Delta G_{gas}+\left(\Delta G_{PB}^{AB}-\Delta G_{PB}^{A}-\Delta G_{PB}^{B}\right)+\left(\Delta G_{SA}^{AB}-\Delta G_{SA}^{A}-\Delta G_{SA}^{B}\right)\\ \;=\Delta G_{gas}+\Delta \Delta G_{PB}+\Delta \Delta G_{SA}\\ \;\approx \Delta E_{gas}-T\Delta S+\Delta \Delta G_{PB}+\Delta \Delta G_{SA}\\ \;=\Delta \left(E_{bond}+E_{angle}+E_{torsion}+E_{vdw}+E_{ele}+\Delta G_{PB}+\Delta G_{SA}\right)-T\Delta S\\ \;=\Delta G_{mmpbsa}-T\Delta S \end{array}$$ (14)

where the superscripts A, B and AB denote receptor, ligand and complex of receptor and ligand respectively.

## Results and Discussion

### The Stable States in the PD-L1s System

These stable binding states can be divided into the initial states (apo PD-L1s dimerization), the final state (holo PD-L1s dimerization), and the intermediate states (stable binding modes in the transformation process of initial states to final state) according to the drug-regulated dimerization process.

#### Initial State 1: Apo PD-L1s Dimerization Mode in 4Z18

All the apo dimerization modes (binding modes) in the crystal structure (PDB ID: 4Z18, 5JDR, 3FN3) are same, and the crystal structure (PDB ID: 4Z18) has the best X-ray diffraction resolution (Chen et al., 2010; Zhang et al., 2017). Thus, the crystal structure (PDB ID: 4Z18) was used as the starting structure for the 150 ns all-atom molecular dynamics (MD) simulation (Alder and Wainwright, 1959; Mccammon et al., 1976; Tuckerman and Martyna, 2000) under the condition described as *Molecular Dynamics Simulations* methods. After visualizing the simulation trajectory using VMD program (Humphrey et al., 1996), it was found that the upper half (IgV domain) of the two PD-L1s had a slight oscillating motion of opening and closing, while the lower half (IgC domain) bound tightly all the time. That is to say, this dimerization mode did not dissociate in the simulation. The root mean square deviation (RMSD) shown in Supplementary Figure S1 was used to quantitatively characterize the stability of the double-chain system (black line: 4Z18_whole_dul) and the single-chain system (red line: 4Z18_whole_sin) respectively. The fluctuation of double-chain RMSD is larger than that of single-chain RMSD, which indicates that there is a certain inter-chain relative motion. This motion conforms to the protein dimer system itself characteristic.

From the last 100 ns equilibrium trajectory, 2,500 frames were uniformly taken out and the molecular mechanics Poisson-Boltzmann surface area method (MM-PBSA) was used to calculate the binding free energy. The $\Delta G_{bind}$ value of 4Z18 whole system is −23.63±10.48kcal/mol (Supplementary Table S1). This indicates that the dimerization mode in crystal structure 4Z18 can be stable thermodynamically. Our results can systematically quantify and verify the inference of existence of apo form PD-L1 dimerization in the previous research (Chen et al., 2010).

The binding sites of PD-L1 with such drugs or the receptor PD-1 are all in the upper IgV domain, while the lower IgC Domain of PD-L1 is an extracellular supporting structure (Chen et al., 2010). Being inspired by the motion mode of “upper loose and lower tight” in our visualization, this 4Z18 dimerization mode was truncated to the upper part 4Z18^U^ and the lower part 4Z18^L^ with the relative position remained unchanged to further clarify the precise binding site of the 4Z18 dimerization mode. The 4Z18^U^ and the 4Z18^L^ were used as the initial structures for 150 ns MD simulation respectively. In 4Z18^U^, the RMSD of double-chain and single-chain structure are shown in the black line and the red line in Supplementary Figure S2A respectively. The significant difference between the two lines reflects the obvious relative motion between the two chains in 4Z18^U^. The same analysis on the stability of 4Z18^L^ shows that the 4Z18^L^ structure has high inter-chain stability, as shown in Supplementary Figure S2B. After visualization of the two trajectories, it was found that the two chains of 4Z18^U^ exhibited obvious opening-closing oscillation in the first 20 ns, and dissociated with significant relative displacement after 60 ns, while the two chains of 4Z18^L^ binding stably in the whole 150 ns trajectory.

In the thermodynamical aspect, the calculated values of $\Delta G_{bind}$ of 4Z18^U^ and 4Z18^L^ are 22.71±8.86kcal/mol (Supplementary Table S2) and −21.62±9.66kcal/mol (Supplementary Table S3) respectively. As our systems are protein-protein interaction (PPI) (Pitre et al., 2008) systems, the standard derivations of the binding free energies $\Delta G_{bind}$ may look a little bit larger than the common protein small molecular systems due to much larger interface conformational flexibility and fluctuation. This phenomenon can also be seen in other similar PPI systems (Nayeem et al., 2017; Shi et al., 2019).

The above results show that the binding site of the 4Z18 dimerization mode is mainly in the lower part of PD-L1, as the upper part has no binding effect in this relative orientation. These results have not been reported in relevant studies, which is maybe mainly because the current studies pay too much attention to the upper half structural domain of PD-L1, and use the truncation model which only preserves the upper part in relevant calculation studies (Zak et al., 2016; Guzik et al., 2017; Skalniak et al., 2017; Basu et al., 2019; Shi et al., 2019; Soremekun et al., 2019; Sun et al., 2019).

#### Final State: Holo PD-L1s Dimerization Mode in 5J89

The structure of BMS-202 is close to the common scaffold of this kind of (2-methyl-3-biphenylyl) methanol drugs and relatively simple and representative in them. Especially, experiments show that BMS-202 also has strong pharmacological activity (Chupak et al., 2017; Chupak and Zheng, 2018). As can be seen, an in-depth study of the action mechanism of BMS-202 is particularly important to improve the efficacy of such kind of drugs in the future. For these reasons, we chose PDB 5J89 (which contains BMS-202) as the object in this study.

The crystal structure (PDB ID: 5J89) was used as the starting structure for the 150 ns all-atom MD simulation under the condition described in *Molecular Dynamics Simulations* methods. As shown in Supplementary Figures S3A,B, the 5J89 is a kind of sandwich structure, with the small-molecular drug BMS-202 entrapped between two PD-L1 monomers.

##### Global Analysis in Protein Level: Stability, Flexibility, and Energy

The double-chain RMSD of the 5J89 dimer is shown by the black line in Supplementary Figure S3A. Considering its relatively large fluctuations, the root mean square fluctuations (RMSF) of each residual in the 150 ns trajectory was calculated, and the results were shown in Supplementary Figure S3B. The RMSF shows that the fluctuations mainly come from the 10-residue-long disordered structure at the end of two chains, which comes from the imperfect of artificial expression of proteins and is not belong to homo sequence. Since it is far away from the binding sites of PD-L1 with drugs and the receptor PD-1, it has little influence on our study. Hence the RMSD of 5J89 homo part (Residue ID: 18-134) was calculated in the 150 ns trajectory, as shown by the red line in Supplementary Figure S3A. The results show that the 5J89 dimerization mode has no obvious inter-chain relative motion. The visualization also showed that this sandwich structure did not dissociate in the whole 150 ns trajectory.

For this 5J89 model, when calculating the binding free energies, there are some considerations needed in terms of geometric symmetry. Because the two PD-L1 monomers are exactly the same sequence, under the ensemble statistics in the case of removal of the BMS-202 molecule, the spatial structures of the two PD-L1 conformations (on average) should be exactly the same, the combination structure of two PD-L1s in the conformational average should be C_2v_ symmetry. However, the average conformation of BMS-202 should have ${\text{σ}}_{\text{V}}$ symmetry.

When the X, Y and Z three-dimensional coordinate system is established for the conformational average structure of 5J89 system and let the XZ plane pass through the plane of the conformational average structure of BMS-202, namely the conformational average binding interface of the two PD-L1 monomers, then the symmetry of the conformational average structure of the two PD-L1 monomers satisfies ${\text{C}}_{2\text{v}}={\text{C}}_{2\text{Z}}$ in this coordinate system, the symmetry of the conformational average structure of the BMS-202 satisfies ${\text{σ}}_{\text{V}}={\text{σ}}_{\text{XZ}}$ , and their matrices in this coordinate system are as follows respectively:

$$C_{2z}=\left[\begin{array}{ccc} -1 & 0 & 0\\ 0 & -1 & 0\\ 0 & 0 & 1 \end{array}\right]\sigma_{xz}=\left[\begin{array}{ccc} 1 & 0 & 0\\ 0 & -1 & 0\\ 0 & 0 & 1 \end{array}\right]C_{2z}=\sigma_{xz}\sigma_{yz}=\sigma_{yz}\sigma_{xz}\text{ where }\sigma_{yz}=\left[\begin{array}{ccc} -1 & 0 & 0\\ 0 & 1 & 0\\ 0 & 0 & 1 \end{array}\right]$$

This means that only when the average conformational structure of BMS-202 also has $\sigma_{yz}$ symmetry, or when the conformational average of the combination structure of two PD-L1 monomers also has $\sigma_{yz}$ symmetry, the binding interface environment between the BMS-202 and the two PD-L1 monomers is completely the same. However, neither of these two conditions can be satisfied. Therefore, according to the above simple analysis of group theory, we need to study the two-binding interface between the BMS-202 and two PD-L1 monomers respectively.

**FIGURE 1:**
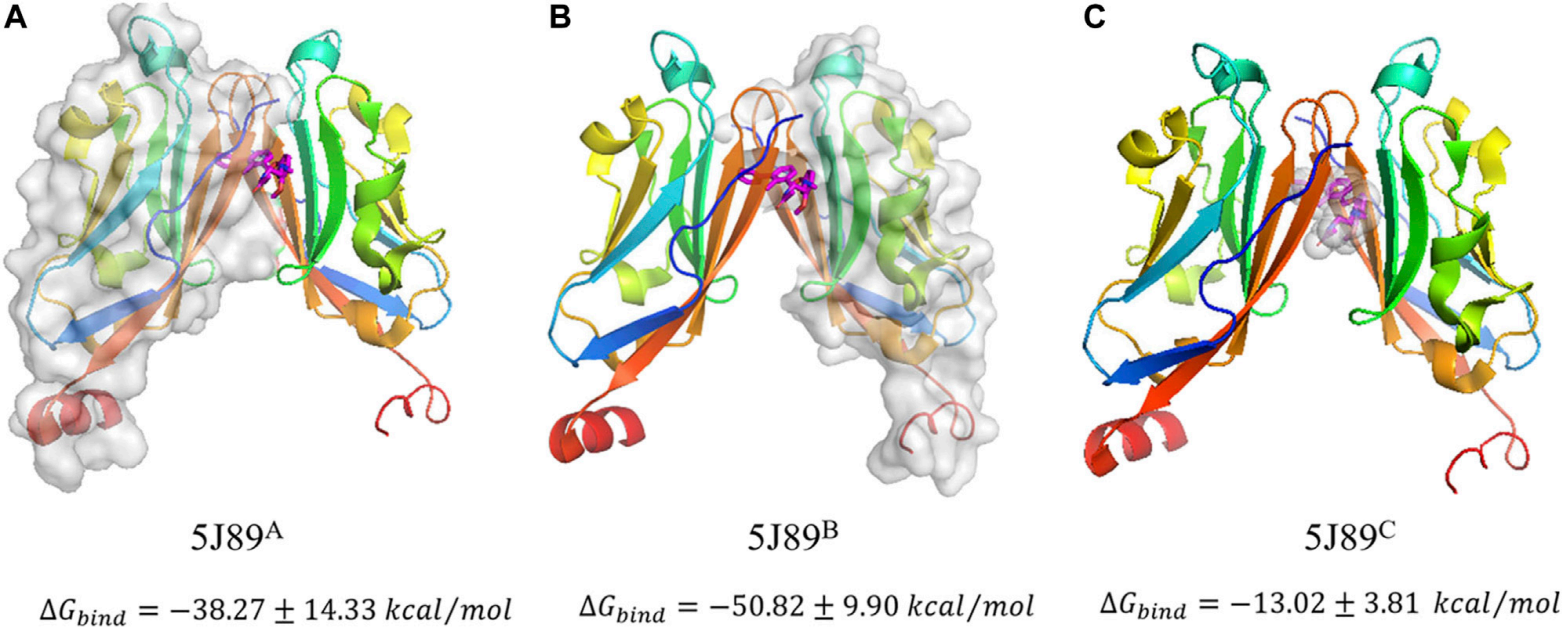
Three kinds of stripping modes of ∆G_bind_ calculation in 5J89 system which are denoted as 5J89^A^ **(A)**, 5J89^B^ **(B)** and 5J89^C^ **(C)** respectively. The gray surfaces represent the stripping interfaces in binding free energy calculations.

For the reasons of symmetry mismatch (between C_2v_ and ${\text{σ}}_{\text{V}}$ ) mentioned above, we calculated $\Delta G_{bind}$ of three kinds of stripping modes (5J89^A^, 5J89^B^ and 5J89^C^) respectively, shown in Figure 1. The detail components of binding free energies are shown in Supplementary Tables S4, S5, S6 respectively.

The negative free energies of three kinds of stripping modes in final state 5J89 have shown the mutual affinity between the three self-assembly blocks (when they stochastic move to the neighbourhood among them), which indicates the feasibility of three-body concerted self-assembly. The results of stripping mode 5J89^A^ and stripping mode 5J89^B^ show that the symmetry mismatch indeed leads to the difference of binding free energy. The $\Delta G_{bind}$ of 5J89^C^ indicates that BMS-202 has a stabilizing effect on PD-L1s dimerization. The $\Delta G_{bind}$ of 5J89^A^ and 5J89^B^ are far higher than the $\Delta G_{bind}$ of 5J89^C^, which indicates that there is a strong protein-protein interaction between the two PD-L1 monomers in 5J89 mode. This is further confirmed in the later (*Three Other Binding States* section). Our binding free energy results provide details in stripping modes, and also consistent with other works about other similar but not the same BMS PD-L1 inhibitors (Shi et al., 2019; Soremekun et al., 2019; Sun et al., 2019).

##### Local Analysis in Residues Level: Energy Decomposition, Key Residue Groups, H-Bonds

**FIGURE 2:**
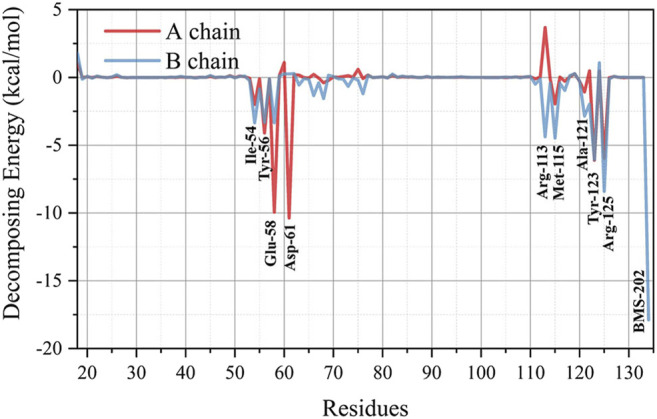
The binding free energy decomposition result of every residue in the 5J89 system.

**FIGURE 3:**
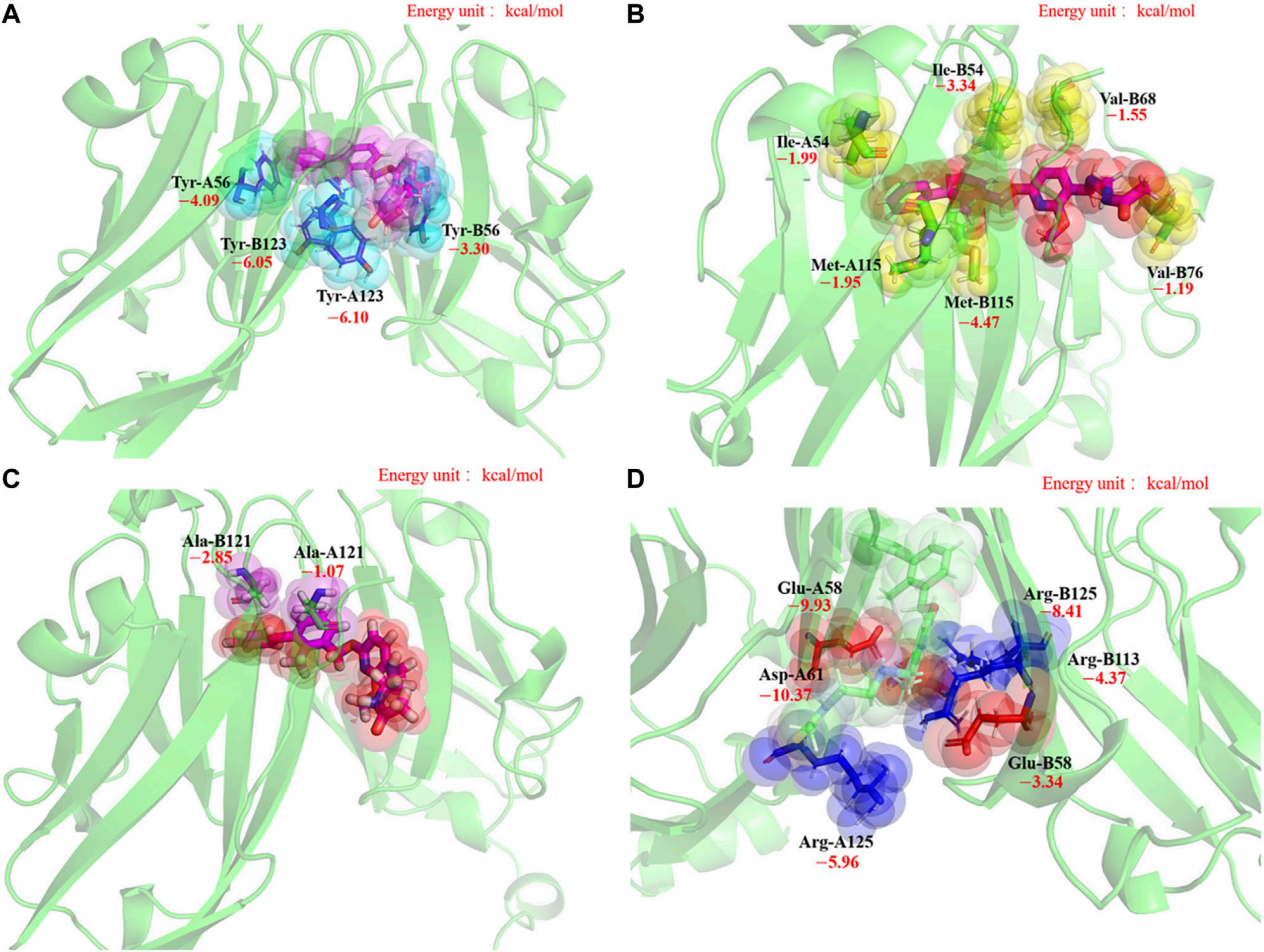
The structures of four key residue groups of the MM-PBSA binding free energy decomposition in the 5J89 system. **(A**–**D)** represent the residue group 1, residue group 2, residue group 3 and residue group 4 respectively.

To further quantify the local structure factors contributing to the stability of the 5J89 dimerization mode from the thermodynamic aspect, the MM-PBSA binding free energy decomposition was performed, and the results are shown in Figure 2. Residues that contribute significantly to the binding stability of the 5J89 dimerization model are labeled in Figure 2. Our energy decomposition results can quantify and verify the previous prediction of key residues in PD-L1 by the Holak. research group (Zak et al., 2016; Guzik et al., 2017). Combined with the simulation trajectory visualization, we can summarize them into the following four key residue groups, as shown in Figure 3.

Group 1: There are four tyrosine residues (Tyr-A56, Tyr-B56, Tyr-A123, Tyr-B123) on the binding surfaces of BMS-202 and PD-L1s, as shown in Figure 3A. We call them “tyrosine sucking disks.” They can oscillate and contact the three hydrophobic aromatic rings of the BMS-202 in various conformations of the NPT ensemble sampling, which can provide stabilization effect by hydrophobic adsorption.

Group 2: Around the BMS-202, there are two isoleucine residues (Ile-A54, Ile-B54), two valine residues (Val-B68, Val-B76), and two methionine residues (Met-A115, Met-B115) branching out six long hydrophobic side chains, as shown in Figure 3B. We call them “hydrophobic shrimp feet,” since a shrimp has three pairs of jaw feet in its mouth. They can oscillate and contact the hydrophobic skeleton of BMS-202 in various conformations of the NPT ensemble sampling, which can provide a hydrophobic chelating stabilization effect.

Group 3: There is a pair of alanine residues (Ala-A121, Ala-B121) clipping the two benzene rings of BMS-202 by σ-π interactions, as shown in Figure 3C. We call them “alanine clips.” Although this structure is small, the steric effect of its hydrophobic geometric complementation plays an important role in preventing BMS-202 shedding.

Group 4: There are three pairs of salt-bridges which anchor to each other by static electricity attraction of opposite charges, that are (−)Glu-A58/(+)Arg-B125, (−)Asp-A61/(+)Arg-B113, (+)Arg-A125/(−)Glu-B58, as shown in Figure 3D. The importance of electrostatic effect in this system is not only reflected in the high contribution of binding free energy decomposition, but also in the fact that electrostatic force is a long-range force, which is extremely important in the self-assembly remote recognition stage.

**FIGURE 4:**
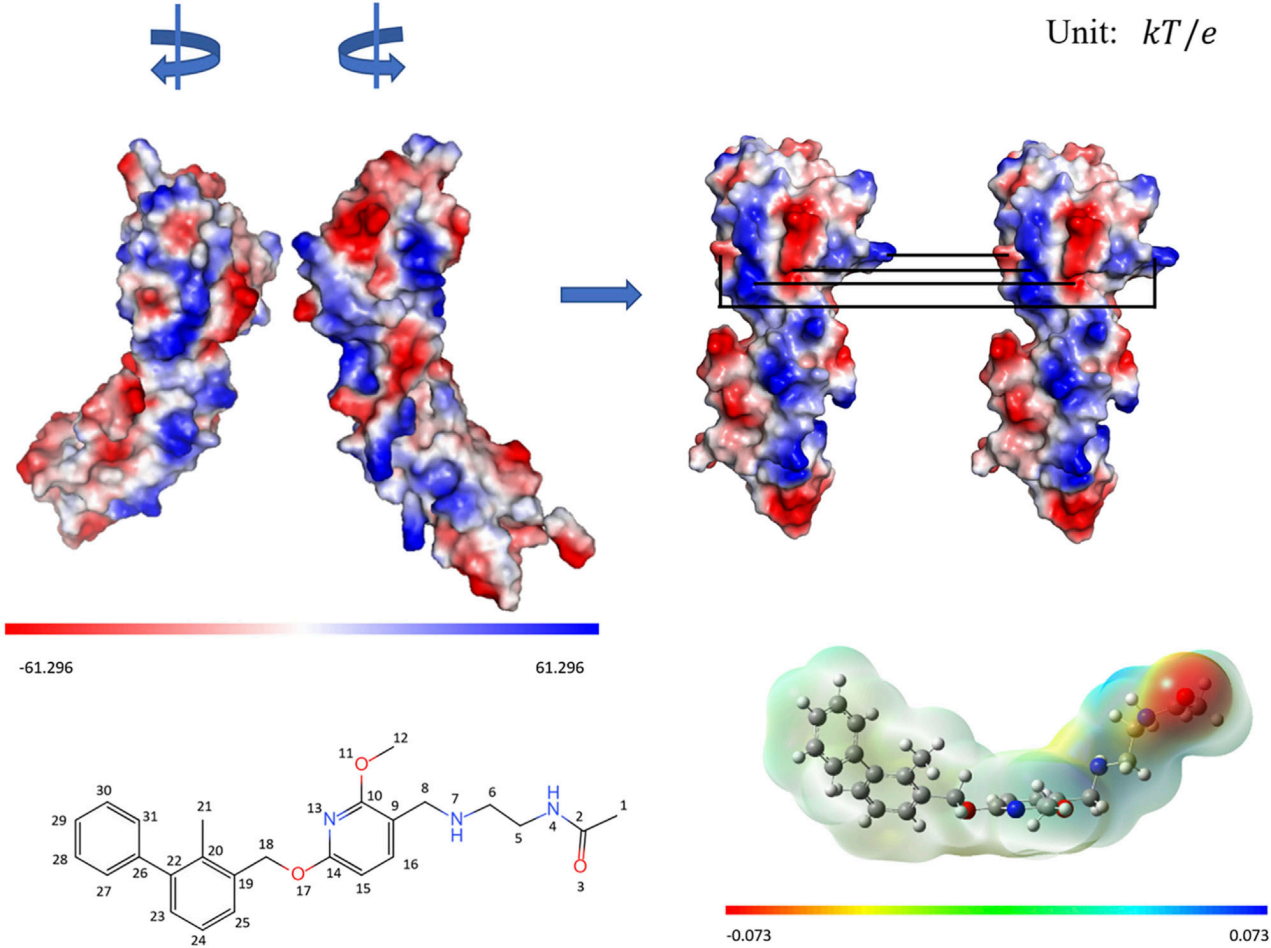
The surface electrostatic potential of PD-L1 and drug molecule BMS-202. The relative orientation of PD-L1s in 5J89 system satisfies the C_2v_ symmetry, and it will satisfy the translation symmetry if we rotate the left monomer 90° clockwise and the right monomer 90° counterclockwise. For ease of viewing, the electrostatic opposite-matching regions of the PD-L1s are linked in pairs by black lines. The surface electrostatic potential and the 2D structure with the atomic numbers of the drug BMS-202 are shown respectively.

To further reveal the electrostatic driving effect, APBS program (Baker et al., 2001) was used to calculate the electrostatic potential on the molecular surface of full-length PD-L1 protein, as shown in Figure 4. The density functional B3LYP was used to calculate the surface electrostatic potential of BMS-202 at the base group level of 6-311+G (2d,p) by Gaussian 09 package (Andersson and Uvdal, 2005; Frisch et al., 2009).

The calculation results of the electrostatic potential of the molecular surfaces show that the C_2v_ symmetry generated by the relative orientation of PD-L1s and the special charge distribution on the surfaces of the PD-L1 upper IgV domains together lead to the electrostatic opposite-matching exactly. Moreover, the characteristic of “alternating electropositive and electronegative of acetylethylenediamine” in the tail of BMS-202 also matches the electrical property on the surface of PD-L1s. This suggests that enhancing the alternating electrostatic potential difference in the tail structure of drug molecules may be more effective in the remote recognition stage of electrostatic driving.

In order to investigate intermolecular H-bonds across binding interface, we conducted statistical analysis of intermolecular H-bonds in 15,000 frames of conformations in our 150 ns trajectory using geometric criteria (Jones et al., 2014) that distance is less than 3.5 Å and angle is greater than 120° and less than 180°. The statistical results are shown in Supplementary Table S7.

The total occupancy of intermolecular H-bond is 11.47, which means that any frame of conformation taken from the NPT ensemble will have 11.47 intermolecular H-bonds on average, which is very considerable in the contribution of intermolecular binding free energy. It is worth mentioning that the residues with high occupancy in the H-bond statistics also contribute highly to the decomposition of binding free energy.

#### Three Other Binding States

The path of a self-assembly system is not single in many cases (Hagan and Chandler, 2006; Grzybowski et al., 2017; Michaels et al., 2018), and therefore the feasibility of other self-assembly path is still worthy to investigate. If the self-assembly process of final state 5J89 is a step by step process, then all the three possible formation processes can be as follows: When we choose a view pose that the acetamide tail of BMS-202 pointing towards us, 1) the PD-L1 binds with BMS-202 as left monomer firstly, 2) the PD-L1 binds with BMS-202 as right monomer firstly, 3) two PD-L1 monomers bind firstly. The three binding states are named as IS^L^, IS^R^ and IS^PP^ respectively and shown in Supplementary Figure S4. Similar to *Global Analysis in Protein Level: Stability, Flexibility, and Energy* section, symmetry mismatch (the C_2v_ group and σ_v_ group) results in different relative binding orientation of the BMS-202 in IS^L^ and IS^R^, which also needs to be studied separately. IS^L^, IS^R^ and IS^PP^ structures were simulated for 150 ns all-atom MD and binding free energies were calculated under the same conditions as before. The results are shown in Supplementary Figures S4A,B,C respectively. The detail components of binding free energies are shown in Supplementary Tables S8, S9, S10 respectively. In the visualization of the MD trajectories, none of the three binding states dissociated. The above results show that all the three binding states can exist stably, hence the step-by-step self-assembly is feasible.

The three stable binding states are discussed in detail as follows.

##### IS^R^ Is More Stable Than IS^L^ as Asymmetry

IS^R^ has −0.14kcal/mol binding free energy superior to IS^L^, which is also consistent with the larger binding free energy when the right PD-L1 monomer stripping from 5J89 as calculated in *Final State: Holo PD-L1s Dimerization Mode in 5J89* section. Combined with the 150 ns trajectories of IS^L^ and IS^R^ and the results of binding free energy decomposition, it can be found that the hydrophobic side chains of Val-B68 ( −1.55kcal/mol ) and Val-B76 ( −1.19kcal/mol ) in IS^R^ are able to chelate the hydrophobic tail of BMS-202. As is shown in Supplementary Figure S5. It is easy to know from the rotational symmetry operation of C_2v_ that the corresponding Val-A68 and Val-A76 in IS^L^ locate on the distal side of the benzene ring head of BMS-202, and they cannot provide the same stabilization as IS^R^ in geometrically and hydrophobic environments. That is to say, asymmetry is what makes the difference.

##### A New Apo Form Dimerization Mode IS^PP^

We discovered a new kind of stable apo form dimerization mode (IS^PP^), which is different from the apo form dimerization 4Z18 reported by previous research (Chen et al., 2010). The result of binding free energy shows that IS^PP^ can exist as a stable dimerization mode in thermodynamic aspect, and compared with the 4Z18 ( $\Delta G_{bind}=-23.63\pm 10.48kcal/mol$ ) calculated in *Initial State 1: Apo PD-L1s Dimerization Mode in 4Z18* section, the IS^PP^ ( $\Delta G_{bind}=-31.15\pm 11.65kcal/mol$ ) is also more stable than 4Z18. From electrodynamics and dispersion aspect, the high ionic strength of solution weakens the polar interactions (ΔE_polar_), but at the same time enhances the nonpolar interactions (ΔE_nonpolar_) (Miller et al., 2012). As shown in Table 1, the ΔE_nonpolar_ in 4Z18 dimer is high and plays a stabilization effect, and the ΔE_polar_ in 4Z18 plays a repulsion effect. While in IS^PP^ the ΔE_polar_ plays a stabilization effect. Therefore, experimental crystallization conditions with high ionic strength may enhance the dimerization stability of 4Z18, but weaken the stability of IS^PP^, making it difficult to find IS^PP^ in the crystallization state.

**TABLE 1:** The binding free energies (kcal mol^−1^) and its components for the dimer 4Z18 and IS^PP^.

	4Z18	IS^PP^
ΔE_vdw_	− 113.96 ± 6.04	− 46.19 ± 6.19
ΔE_ele_	− 456.16 ± 38.18	− 280.86 ± 50.21
ΔG_PB_	487.86 ± 36.40	264.66 ± 42.01
ΔG_SA_	− 14.65 ± 0.42	− 6.00 ± 0.39
ΔE_polar_ ^a^	31.70 ± 52.75	− 16.20 ± 65.46
ΔE_nonpolar_ ^b^	− 128.61 ± 6.05	− 52.19 ± 6.20
ΔG_mmpbsa_ ^c^	− 96.91 ± 9.58	− 68.40 ± 11.22
TΔS	− 73.28 ± 4.24	− 37.25 ± 3.12
ΔG_bind_	− 23.63 ± 10.48	− 31.15 ± 11.65

a ΔE_polar_ = ΔE_ele_+ΔG_PB_.

b ΔE_nonpolar_ = ΔE_vdw_+ΔG_SA_.

c ΔG_mmpbsa_ = ΔE_vdw_+ΔE_ele_+ΔG_PB_+ΔG_SA_ = ΔE_polar_+ΔE_nonpolar_.

##### BMS-202 Can Stabilize the IS^PP^

Comparing the RMSD analysis of IS^PP^ with 5J89 (Supplementary Figures S3A, S4C), indicates that drug BMS-202 did stabilize the PD-L1s dimerization system.

To further understand the influence of drug insertion on the stability of the PD-L1 dimer, the RMSF of homo residues of 5J89 and IS^PP^ in the 150 ns MD trajectories were calculated respectively. From Supplementary Figure S6, we can see that the insertion of BMS-202 can stabilize the entire dimer structure by reducing the motion flexibility of the residue conformations in IS^PP^.

**FIGURE 5:**
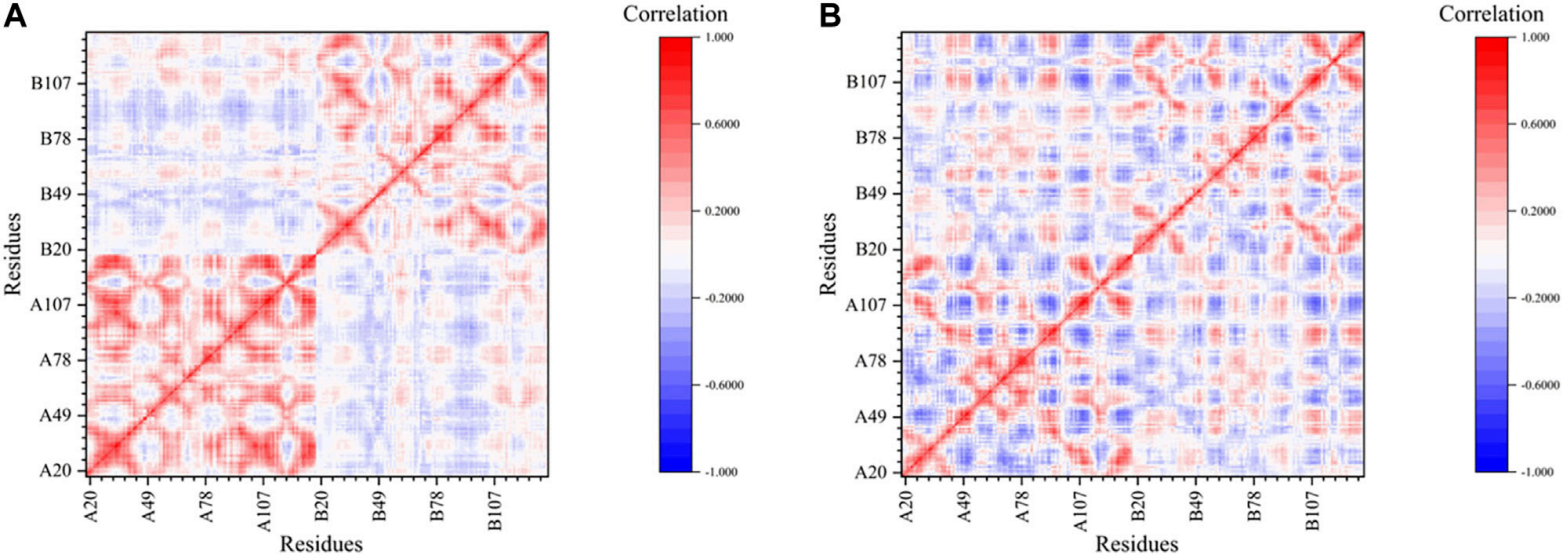
The dynamic cross-correlation matrix (DCCM) of 5J89 system **(A)** and IS^PP^ system **(B)**.

The dynamic cross-correlation matrix (DCCM) (Hunenberger et al., 1995) was calculated to discuss the motion correlation in the trajectories of the 5J89 system and the IS^PP^ system (Figure 5). Each matrix element is the motion correlation coefficient between the two residues, and its value is between −1 and 1.

It can be clearly seen from Figure 5A that this matrix can be divided into four sub-blocks, among them two deep red sub-blocks arranged along the main diagonal show the strong-positive-correlation inner-chain motion of the two chains in 5J89 respectively, which is a kind of integral motion within each chain. In contrast, there are no diagonal sub-blocks in Figure 5B, as the integral motion correlation of the chains in IS^PP^ is weaker. The reason of difference may be due to that the drug insertion makes the inner-chain motion correlation of the residues stronger, and the random vibrations of the atoms in the chain change into the overall consistent motions. That is to say, the BMS-202 can stabilize the IS^PP^. Our results reveal the stabilization effect produced by BMS-202 from different perspective, consistent with experiment works about other similar BMS inhibitors (Zak et al., 2016; Guzik et al., 2017; Shi et al., 2019; Soremekun et al., 2019; Sun et al., 2019).

### Kinetics of Inter-State Transformation

#### Investigate the Kinetics of “BMS-202 Inserting Process” by ASMD

In order to investigate the kinetic feasibility of “BMS-202 inserting process,” adaptive steered molecular dynamics (ASMD) simulation (Ozer et al., 2010; Ozer et al., 2012) was performed to pull BMS-202 out slowly along the cavity (Supplementary Figure S7) in a near equilibrium state to simulate the reverse process of drug insertion. The cavity formed upon the dimerization interface by the native cleft of PD-L1 monomer is important for the drug stabilization (Zak et al., 2016; Guzik et al., 2017).

On the basis of the Jarzynski’s equality (Ozer et al., 2010; Ozer et al., 2012), the potential of mean forces (PMF) profile (Supplementary Figure S8) displayed the free energy changes of the system for the departure of the BMS-202 from the cavity.

Based on the PMF results of ASMD and the previous free energy results of MM-PBSA, we constructed a free energy surface profile (Supplementary Figure S9A) to describe the BMS-202 insertion process. It’s worth noting that, the final PMF value of the steering process is 33.10kcal/mol , containing not only the stabilizing energy 12.82kcal/mol by the drug insertion in *Final State: Holo PD-L1s Dimerization Mode in 5J89* section, but also the resistance energy by the residue side chains in the cavity. Thus, the energy barrier generated by the geometric resistance of the protein cavity is approximately 33.10−12.82=20.28kcal/mol . From the free energy surface profile (Supplementary Figure S9A), the free energy of the transition state in the “BMS-202 inserting process” is −10.87kcal/mol . That is to say, the “BMS-202 inserting process” is feasible in the kinetic aspect, as its transition state free energy is not higher than the transition state of three-body separation ( 0kcal/mol ).

In addition, the following phenomena are observed in the ASMD trajectory: During the pull-out process, the biphenyl structure of the head of BMS-202 is significantly twisted, which shows that the angle between the two planes of the biphenyl rings is gradually reduced from the initial of 60° to about 0° degrees to adapt to the protein cavity. Moreover, the side-chains conformations of the “hydrophobic shrimp feet,” “alanine clips” and “tyrosine sucking disks” of the protein interface are also twisted by the displacement of BMS-202. All of these phenomena reveal the key interactions between BMS-202 and PD-L1 dimers from another perspective. In combination with the previous analysis in *BMS-202 Can Stabilize the IS^PP^* section, it is reasonable to conclude that the insertion of BMS-202 produces a hydrophobic geometric complementary stabilization.

Compositing the transition state of “BMS-202 inserting process” ( −10.87kcal/mol ), the transition state of “three-body separation” ( 0kcal/mol ) and the previous free energy results of all the stable PD-L1 dimerization modes by MM-PBSA, we constructed a integral free energy surface (Supplementary Figure S9B) to investigate the integral inter-state transformation kinetics in this multiple-states system.

#### Investigate the Kinetic Characteristics of Inter-State Transformation by GITR Theory

**FIGURE 6:**
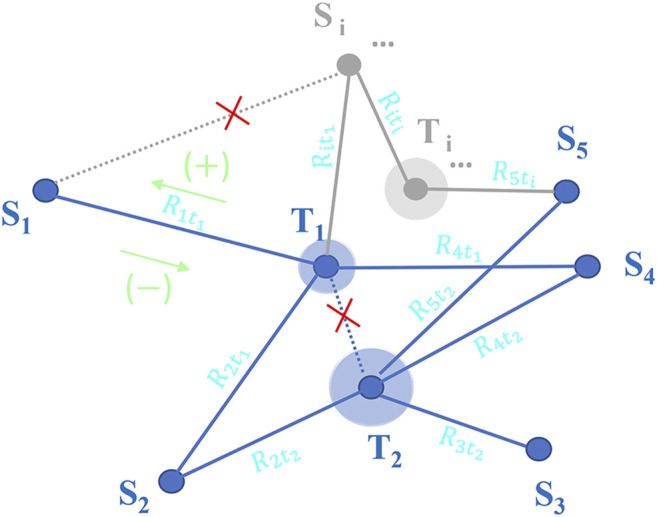
Each stable state S refers to the stable polymerization state formed by the aggregation of ${\text{ν}}_{\text{s}}$ self-assembly monomers. Each transition state T refers to the Gibbs free energy maximum point on the transformation path. There is no direct linking between any two stable states or any two transition states. For each transition state, we add a disk to distinguish it from the stable state, and the size of the disk is also measured by the relative Gibbs free energy. The $R_{it_{i}}$ refers the transformation rate of stable state S_i_ via transition state T_i_ with the minus sign represents a decrease in concentration and the plus sign represents an increase in concentration. For the generality of the theory, the potential stable states and transition states are marked in gray. The ${\text{C}}_{\text{s}}$ which refers the concentration of stable state S is counted by polymerization cluster. The ${\text{C}}_{0}$ refers the total analytical concentration of self-assembly monomer in whole system. The ${\text{ G}}_{\text{s}}$ and ${\text{G}}_{{\text{t}}_{\text{i}}}$ are Gibbs free energies of the monomers in stable state S and transition state $t_{i}$ respectively. The Gibbs free energy of the free monomer state is set as the zero point of whole energy scale.

**FIGURE 7:**
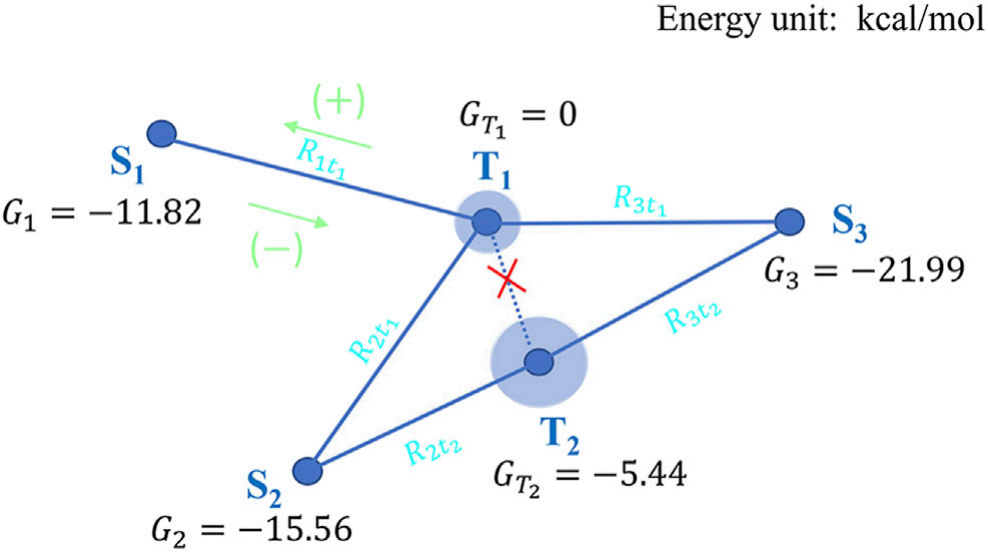
The representation of the PD-L1 multiple-states system according to GITR theory.

The inter-state transformation of three stable dimerization modes in the PD-L1 system can constitute a network (Figure 7), in which the existence of each dimerization mode in the system will affect the concentration and transformation rate of a specific dimer mode in the network. For this reason, the inter-state transformation kinetics is different from the traditional simple reaction kinetics problems. This kind of multiple-states self-assembly networks (Figure 6) also exist in other systems (Hagan and Chandler, 2006; Rha et al., 2009; Pashuck and Stupp, 2010; Adamcik et al., 2011; Martinez-Avila et al., 2012; Grzybowski et al., 2017; Michaels et al., 2018; Dai et al., 2019). We attempt to provide a unified solution—generalized inter-state transformation rate (GITR) theory to this kind of problem.

##### Generalized Inter-State Transformation Rate Theory

For a complex self-assembly network containing any finitely many stable states (self-assembly modes) and any finitely many transition states (the Gibbs free energy maximum point on the transformation path), with arbitrary links between them, the transformation rates between self-assembly states in the network can be calculated by GITR theory. Our GITR theory has the following three basic hypotheses. To self-assembly system, they are rational and physical.

1) Single transition state hypothesis: It is reasonable because if a transformation between two stable states via two transition states (Gibbs free energy maximum points), we can find and insert a new stable state between the two transition states as a new addition.
2) The transition states are high energy and low concentration.
3) Inter-state transformation of the self-assembly system follows first-order kinetics. It is reasonable in many inter-state transformation problems of complex self-assembled systems (Odde et al., 1995; Cheng et al., 2011; Saric et al., 2016; Michaels et al., 2018). Because in many cases, the rate-determining step of the transformation process from state $S_{1}$ to state $S_{2}$ of a self-assembly system depends on the energy obtaining of the monomer in state $S_{1}$ or even the conformation transformation to become the activated monomer in the $S_{1}$ - $S_{2}$ transition state (Cooper et al., 1983; Frieden, 1985; Kentsis and Borden, 2004; Jiang and Prentiss, 2014; Fu et al., 2015; Zierenberg et al., 2017). As a result, the rate-determining step is not intuitively the multi-monomer aggregation step.

###### Differential Rate Theory in the Network

From the classical transition state theory (CTST) (Eyring, 1935; Ross and Vlad, 1999; Fernandez-Ramos et al., 2006), the rate constant of elemental reaction is determined by the activation Gibbs free energy. As mentioned above, since the rate of inter-state transformation of many complex self-assembly systems is determined by the energy activation step of the monomer, the transformation rate from stable state S to transition state $t_{i}$ depends on the difference of monomer Gibbs free energy between the transition state $t_{i}$ and the stable state S. The transformation from the transition state $t_{i}$ to each directly linked stable state S is non-barrier process, so we propose the concept of linking fraction matrix $L_{f}$ . The s th row and $t_{i}$ th column element of $L_{f}$ represents the distribution ratio from the transition state $t_{i}$ to the directly linked stable state S. We will figure out the elements of linking fraction matrix $L_{f}$ later. Since the instantaneous transformation at a certain moment is an infinitesimal compared with $C_{s}$ , the transformation paths of a given stable state to any finitely many transition states will not affect each other. The transformation from stable state S to transition state $t_{i}$ is artificially selected to be negative and from transition state $t_{i}$ to stable state S to be positive. Thus, for any two directly linked states S and $t_{i}$ in the network, we get the corresponding path rate

$$R_{st_{i}}=-\frac{k_{B}T}{h}A_{t_{i}s}e^{-\frac{G_{t_{i}}-G_{s}}{N_{A}k_{B}T}}\times C_{s}+\sum_{s^{\prime }}L_{f_{st_{i}}}\frac{k_{B}T}{h}A_{t_{i}s^{\prime }}e^{-\frac{G_{t_{i}}-G_{s^{\prime }}}{N_{A}k_{B}T}}\frac{\nu_{s^{\prime }}}{\nu_{s}}C_{s^{\prime }}$$ (15)

where the ${\text{C}}_{\text{s}}$ which refers the concentration of stable state S is counted by polymerization cluster, $\nu_{s}$ is the number of monomers in a cluster S, $N_{A}$ is Avogadro’s constant, $k_{B}$ is Boltzmann constant, h is Planck’s constant. All dummy indices summed over are marked with an apostrophe. Considering that in many cases, the activated monomer needs a dissociation process from the previous stable state or even a conformation transformation process, the rate difference between each path may be generated. (Cooper et al., 1983; Frieden, 1985; Kentsis and Borden, 2004; Jiang and Prentiss, 2014; Zierenberg et al., 2017) We introduce a dimensionless frequency factor $A_{t_{i}s}$ in the above formula to correct this process.

Let $k_{t_{i}s}=\frac{k_{B}T}{h}A_{t_{i}s}$ and $R=N_{A}k_{B}$ , then

$$R_{st_{i}}=-k_{t_{i}s}e^{-\frac{G_{t_{i}}-G_{s}}{RT}}\times C_{s}+\sum_{s^{\prime }}L_{f_{st_{i}}}k_{t_{i}s^{\prime }}e^{-\frac{G_{t_{i}}-G_{s^{\prime }}}{RT}}\frac{\nu_{s^{\prime }}}{\nu_{s}}C_{s^{\prime }}$$ (16)

Sum $R_{st_{i}}$ over all the index $t_{i}$ that directly linked to the state S, then the following state rate $R_{s}$ represents the concentration change rate of stable state S:

$$R_{s}=\underset{t_{i}^{\prime }}{\sum }R_{st_{i}^{\prime }}=\underset{t_{i}^{\prime }}{\sum }-k_{t_{i}^{\prime }s}e^{-\frac{G_{t_{i}^{\prime }}-G_{s}}{RT}}\times C_{s}+\sum_{t_{i}^{\prime }}\sum_{s^{\prime }}\frac{1}{\nu_{s}}L_{f_{st_{i}^{\prime }}}k_{t_{i}^{\prime }s^{\prime }}e^{-\frac{G_{t_{i}^{\prime }}-G_{s^{\prime }}}{RT}}\nu_{s^{\prime }}C_{s^{\prime }}$$ (17)

Now let’s calculate the path rate at equilibrium concentration. In order to obtain the path rate under equilibrium condition, we should firstly obtain the equilibrium concentration $C_{s}^{eq}$ of the assembly state S. For the generality of the theory, we get $C_{s}^{eq}$ by the probability $P_{s}$ that the monomers exist in the assembly state S at equilibrium.

Considering the grand canonical ensemble of self-assembly monomers under chemical potential μ, its grand partition function is

$$\Xi \left(V,T,\mu \right)=\sum_{N}\sum_{j}e^{-\frac{E_{N,j}}{k_{B}T}}e^{\frac{\mu N}{k_{B}T}}$$ (18)

where N is the number of monomers in the copy of the system with volume V, j is the j th quantum state of the system copy with volume V and number of monomers N, and $E_{N,j}$ is the energy of the j th quantum state of the system copy with number of monomers N.

Sum over the index j for a particular N, then the canonical partition function Q(N,V,T) of the system can be obtained. After substituting it in, the following equation can be obtained:

$$\Xi \left(V,T,\mu \right)=\sum_{N}^{\infty }Q\left(N,V,T\right)e^{\frac{\mu N}{k_{B}T}}$$ (19)

Since the self-assembly monomer can be regarded as an indistinguishable delocalized particle, replace Q(N,V,T) by the canonical partition function of monomer q(V,T) , then

$$\Xi \left(V,T,\mu \right)=\sum_{N}^{\infty }\frac{q^{N}\left(V,T\right)}{N!}e^{\frac{\mu N}{k_{B}T}}$$ (20)

Sum over the index N, we get

$$\Xi \left(V,T,\mu \right)=\sum_{N}^{\infty }\frac{{\left(q\left(V,T\right)e^{\frac{\mu }{k_{B}T}}\right)}^{N}}{N!}=exp\left[q\left(V,T\right)e^{\frac{\mu }{k_{B}T}}\right]$$ (21)

Calculate the grand canonical ensemble average for the number of monomers N:

$$\langle N\rangle =\frac{\partial ln\Xi \left(V,T,\mu \right)}{\partial \frac{\mu }{k_{B}T}}=q\left(V,T\right)e^{\frac{\mu }{k_{B}T}}$$ (22)

Since the volume V of all copies of the system in the grand canonical ensemble is constant, this indicates that the monomer concentration M is proportional to $e^{\frac{\mu }{k_{B}T}}$ :

$$M=\frac{\langle N\rangle }{N_{A}V}\propto e^{\frac{\mu }{k_{B}T}}$$ (23)

We can write this relation in a more common form, for example, when self-assembly equilibrium is reached

$$N_{A}\mu^{eq}=N_{A}\mu_{s}^{{}^{\circ}}+RTln\frac{M_{s}^{eq}}{M^{{}^{\circ}}}$$ (24)

where $M_{s}^{eq}$ and $M^{{}^{\circ}}$ are respectively the concentration of monomer in self-assembly state S at equilibrium and the concentration of monomer in standard state. $\;\mu^{eq}$ and $\mu_{s}^{{}^{\circ}}$ are respectively the chemical potential of monomer at equilibrium and the chemical potential of monomer in the standard state of self-assembly state S.

Therefore, the monomer concentration in each assembly state S at equilibrium is

$$M_{s}^{eq}=M^{{}^{\circ}}e^{\frac{N_{A}\left(\mu^{eq}-\mu_{s}^{{}^{\circ}}\right)}{RT}}$$ (25)

As a result, the probability $P_{s}$ that the monomers exist in the assembly state S at equilibrium is

$$P_{s}=\frac{M_{s}^{eq}}{\sum_{s^{\prime }}M_{s^{\prime }}^{eq}}=\frac{e^{-\frac{N_{A}\mu_{s}^{{}^{\circ}}}{RT}}}{\sum_{s^{\prime }}e^{-\frac{N_{A}\mu_{s\prime }^{{}^{\circ}}}{RT}}}=\frac{e^{-\frac{N_{A}\mu^{{}^{\circ}}+\frac{1}{\nu_{s}}\sum \Delta G_{s}^{bind}}{RT}}}{\sum_{s^{\prime }}e^{-\frac{N_{A}\mu^{{}^{\circ}}+\frac{1}{\nu_{s\prime }}\sum \Delta G_{s^{\prime }}^{bind}}{RT}}}=\frac{e^{-\frac{\frac{1}{\nu_{s}}\sum \Delta G_{s}^{bind}}{RT}}}{\sum_{s^{\prime }}e^{-\frac{\frac{1}{\nu_{s\prime }}\sum \Delta G_{s^{\prime }}^{bind}}{RT}}}=\frac{e^{-\frac{G_{s}}{RT}}}{\sum_{s^{\prime }}e^{-\frac{G_{s^{\prime }}}{RT}}}$$ (26)

where the $G_{s}=\frac{1}{\nu_{s}}\sum \Delta G_{s}^{bind}$ means using the average of $\Delta G_{s}^{bind}$ of step-by-step stripping processes as the measurement of average monomer free energy $G_{s}$ in state S.

For simplicity, we let

$$Z=\sum_{s^{\prime }}e^{-\frac{G_{s^{\prime }}}{RT}}$$ (27)

Note that, Z is constant for a given self-assembly system.

Therefore, we get

$$C_{s}^{eq}=\frac{1}{\nu_{s}}P_{s}C_{0}=\frac{1}{\nu_{s}}\frac{1}{Z}e^{-\frac{G_{s}}{RT}}C_{0}$$ (28)

As a result, the path rate at equilibrium concentration is

$$\begin{array}{l} {\left[R_{st_{i}}\right]}_{eq}=-k_{t_{i}s}e^{-\frac{G_{t_{i}}-G_{s}}{RT}}\times \frac{1}{\nu_{s}}\frac{1}{Z}e^{-\frac{G_{s}}{RT}}C_{0}+\underset{s^{\prime }}{\sum }\frac{1}{\nu_{s}}\nu_{s^{\prime }}L_{f_{st_{i}}}k_{t_{i}s^{\prime }}e^{-\frac{G_{t_{i}}-G_{s^{\prime }}}{RT}}\frac{1}{\nu_{s^{\prime }}}\frac{1}{Z}e^{-\frac{G_{s^{\prime }}}{RT}}C_{0}\\ =-k_{t_{i}s}e^{-\frac{G_{t_{i}}-G_{s}}{RT}}\times \frac{1}{\nu_{s}}\frac{1}{Z}e^{-\frac{G_{s}}{RT}}C_{0}+\frac{1}{\nu_{s}}L_{f_{st_{i}}}\left(\underset{s^{\prime }}{\sum }k_{t_{i}s^{\prime }}\right)\frac{1}{Z}e^{-\frac{G_{t_{i}}}{RT}}C_{0} \end{array}$$ (29)

Since equilibrium has been reached and there is no net flow, the calculated path rate at equilibrium concentration should be 0:

$$k_{t_{i}s}=L_{f_{st_{i}}}\left(\sum_{s^{\prime }}k_{t_{i}s^{\prime }}\right)$$ (30)

i.e.,

$$L_{f_{st_{i}}}=k_{t_{i}s}/\sum_{s^{\prime }}k_{t_{i}s^{\prime }}$$ (31)

We can now see the physical meaning of linking fraction matrix $L_{f}$ : In a rough approximation, all the frequency factors $A_{t_{i}s}$ can be equal. So all of the $k_{t_{i}s}$ are equal to, let’s say, k. Then the distribution ratio of the transition state $t_{i}$ to each stable state is equal to 1/n, where n is the number of stable states directly linked to the transition state $t_{i}$ . This is consistent well with the physical images. Because the transformation from the transition state $t_{i}$ to each directly linked stable state S is non-barrier process and all the $k_{t_{i}s}$ now is equal to k, and the $k_{t_{i}s}$ contains the information of dissociation frequency of reaction path, it means that all the dissociation frequencies of paths are equal, then the transformations of transition state $t_{i}$ to the direct linked stable states should be equiprobable, which are equal to 1/n. For the simple example shown in the figure above, its $L_{f}$ matrix should be

$$L_{f}={\left[\begin{array}{ccc} 1/4 & 0 & 0\\ 1/4 & 1/4 & 0\\ 0 & 1/4 & 0\\ 1/4 & 1/4 & 0\\ 0 & 1/4 & 1/2\\ 1/4 & 0 & 1/2 \end{array}\right]}^{n_{s}\times n_{t}}$$ (32)

where $n_{s}=6$ and $n_{t}=3$ .

In the more precise case, $L_{f_{st_{i}}}=k_{t_{i}s}/\sum_{s^{\prime }}k_{t_{i}s^{\prime }}$ represents the transformation proportion of the transition state $t_{i}$ to the directly linked stable state S. It is determined by the probability of path selection from all the $t_{i}$ directly linked paths and the dissociation frequency along the reaction path together. For the simple example shown in the figure above, its $L_{f}$ matrix should accurately be

$$L_{f}={\left[\begin{array}{ccc} k_{t_{1}1}/\sum_{s^{\prime }=1}^{6}k_{t_{1}s^{\prime }} & 0 & 0\\ k_{t_{1}2}/\sum_{s^{\prime }=1}^{6}k_{t_{1}s^{\prime }} & k_{t_{2}2}/\sum_{s^{\prime }=1}^{6}k_{t_{2}s^{\prime }} & 0\\ 0 & k_{t_{2}3}/\sum_{s^{\prime }=1}^{6}k_{t_{2}s^{\prime }} & 0\\ k_{t_{1}4}/\sum_{s^{\prime }=1}^{6}k_{t_{1}s^{\prime }} & k_{t_{2}4}/\sum_{s^{\prime }=1}^{6}k_{t_{2}s^{\prime }} & 0\\ 0 & k_{t_{2}5}/\sum_{s^{\prime }=1}^{6}k_{t_{2}s^{\prime }} & k_{t_{3}5}/\sum_{s^{\prime }=1}^{6}k_{t_{3}s^{\prime }}\\ k_{t_{1}6}/\sum_{s^{\prime }=1}^{6}k_{t_{1}s^{\prime }} & 0 & k_{t_{3}6}/\sum_{s^{\prime }=1}^{6}k_{t_{3}s^{\prime }} \end{array}\right]}^{n_{s}\times n_{t}}$$ (33)

The single-direction interaction path rate at the equilibrium concentration can be calculated as follows:

$$\begin{array}{l} {\left[R_{st_{i}}\right]}_{\left||eq|\right|}=k_{t_{i}s}e^{-\frac{G_{t_{i}}-G_{s}}{RT}}\times \frac{1}{\nu_{s}}\frac{1}{Z}e^{-\frac{G_{s}}{RT}}C_{0}=L_{f_{st_{i}}}\left(\sum_{s^{\prime }}k_{t_{i}s^{\prime }}\right)\frac{1}{\nu_{s}}\frac{1}{Z}e^{-\frac{G_{t_{i}}}{RT}}C_{0}\\ =k_{t_{i}s}\frac{1}{\nu_{s}}\frac{1}{Z}e^{-\frac{G_{t_{i}}}{RT}}C_{0}=\frac{k_{B}T}{h}A_{t_{i}s}\frac{1}{\nu_{s}}\frac{1}{Z}e^{-\frac{G_{t_{i}}}{RT}}C_{0} \end{array}$$ (34)

When we calculate the single-direction interaction path rate at equilibrium concentration in monomer form, it should be

$${\left[R_{st_{i}}^{mon}\right]}_{\left||eq|\right|}=\frac{k_{B}T}{h}A_{t_{i}s}\frac{1}{Z}e^{-\frac{G_{t_{i}}}{RT}}C_{0}$$ (35)

The above formula shows that the single-direction interaction path rate at equilibrium (in monomer form) is only related to the free energy of monomer in the transition state $t_{i}$ and the frequency factor $A_{t_{i}s}$ of the corresponding path, but has no relation to the energy of stable state S. Again, this adheres to our intuition physics intuitive. The more stable the state with lower energy has a larger population, but it needs to climb a higher activation energy barrier. The transition state $t_{i}$ acts as the hinge of rate control in the network, and its monomer free energy $G_{t_{i}}$ determines the probability of its occurrence in the system, so it determines the single-direction interaction path rate of the network.

###### Integral Time-Dependent Evolution of Concentrations in the Network

Since

$$R_{s}=\sum_{t_{i}^{\prime }}R_{st_{i}^{\prime }}=\sum_{t_{i}^{\prime }}-k_{t_{i}^{\prime }s}e^{-\frac{G_{t_{i}^{\prime }}-G_{s}}{RT}}C_{s}+\sum_{t_{i}^{\prime }}\sum_{s^{\prime }}\frac{1}{\nu_{s}}L_{f_{st_{i}^{\prime }}}k_{t_{i}^{\prime }s^{\prime }}e^{-\frac{G_{t_{i}^{\prime }}-G_{s^{\prime }}}{RT}}\nu_{s^{\prime }}C_{s^{\prime }}$$ (36)

and $\frac{dC_{s}}{dt}=R_{s}$ , write the above equation in vector and matrix form:

$$\begin{array}{l} \frac{dC}{dt}=\left[\begin{array}{ccc} \sum_{t_{i^{\prime }}}-k_{t_{i}^{\prime }1}e^{\frac{G_{t_{i}^{\prime }}-G_{1}}{RT}} & \cdots & 0\\ \vdots & \ddots & \vdots \\ 0 & \cdots & \sum_{t_{i^{\prime }}}-k_{t_{i}^{\prime }n}e^{-\frac{G_{t_{i}^{\prime }}-G_{n}}{RT}} \end{array}\right]\left[\begin{array}{c} C_{1}\\ \vdots \\ C_{n} \end{array}\right]\\ +\left[\begin{array}{ccc} \frac{1}{\nu_{1}} & \cdots & 0\\ \vdots & \ddots & \vdots \\ 0 & \cdots & \frac{1}{\nu_{n}} \end{array}\right]\left[L_{f_{st_{i}}}\right]\left[k_{t_{i}s}e^{-\frac{G_{t_{i}}-G_{s}}{RT}}\right]\left[\begin{array}{ccc} \nu_{1} & \cdots & 0\\ \vdots & \ddots & \vdots \\ 0 & \cdots & \nu_{n} \end{array}\right]\left[\begin{array}{c} C_{1}\\ \vdots \\ C_{n} \end{array}\right] \end{array}$$ (37)

In fact, both $k_{t_{i}s}$ and the linking fraction matrix element $L_{f_{st_{i}}}$ record the topology information of the self-assembly network, because $k_{t_{i}s}$ and $L_{f_{st_{i}}}$ are 0 when the transition state $t_{i}$ and stable state S are not directly linked. After the above formula is simplified and combined, the following equation can be obtained:

$$\frac{dC}{dt}={\left[{\left(\left(\sum_{t_{i}^{\prime }}-k_{t_{i}^{\prime }s}e^{-\frac{G_{t_{i}^{\prime }}-G_{s}}{RT}}\right)\delta_{sj}+\frac{\nu_{j}}{\nu_{s}}L_{f_{st_{i}^{\prime }}}{\hat{\Delta G}}_{t_{i}^{\prime }j}\right)}_{sj}\right]}^{n_{s}\times n_{s}}C$$ (38)

where $\hat{\Delta G}$ represents the matrix $\;{\left[{\left(k_{t_{i}s}e^{-\frac{G_{t_{i}}-G_{s}}{KT}}\right)}_{t_{i}s}\right]}^{n_{t}\times n_{s}}$ and $\delta_{sj}$ is Kronecker notation.

Because we’ve already written the above formula in this $\frac{dC}{dt}=\hat{A}C$ form, C has to have a particular solution $C=\xi e^{\lambda t}$ . After substituting it in, the following equation can be obtained:

$$\left(\lambda \hat{E}-\hat{A}\right)\xi e^{\lambda t}=0$$ (39)

where $\hat{E}$ is the identity matrix. Since $C=\xi e^{\lambda t}\neq 0$ , ξ is the eigenvector of matrix $\hat{A}$ corresponding to the eigenvalue λ.

According to the superposition of the solutions, the general solution is

$$C=\sum_{i}z_{i}\xi_{i}e^{\lambda_{i}t}$$ (40)

where, $z_{i}$ is determined by the initial concentration condition, $\xi_{i}$ is the eigenvector of matrix ${\left[{\left(\left(\sum_{t_{i}^{\prime }}-k_{t_{i}^{\prime }s}e^{-\frac{G_{t_{i}^{\prime }}-G_{s}}{RT}}\right)\delta_{sj}+\frac{\nu_{j}}{\nu_{s}}L_{f_{st_{i}^{\prime }}}{\hat{\Delta G}}_{t_{i}^{\prime }j}\right)}_{sj}\right]}^{n_{s}\times n_{s}}$ corresponding to the eigenvalue $\lambda_{i}$ .

According to the integral results of the time-dependent evolution of the network, the concentration change rates of different stable states are determined by the same set of eigenvalues of the dynamic matrix $\hat{A}$ , and a variety of $\lambda_{i}$ can be regarded as a variety of time evolution scale factors. All the stable states in the network evolute in the same power law, the only difference is the front multiplicative factors which are the components of eigenvector $\xi_{i}$ . These front multiplicative factors can be regarded as the weight coefficients of each time scale in different stable states. This is also an interesting finding. In addition, if we let the matrix $\left[k_{t_{i}s}\right]$ evolve with the vector C and update by appropriate steps, the GITR theory would also be able to solve more complex non-linear kinetics problem.

##### Application of the GITR Theory to PD-L1 System

As a specific application of the GITR theory, we can now analyze the inter-state transformation kinetics in PD-L1 system. In Figure 7, the S_1_, S_2_ and S_3_ denote the initial state 1 (4Z18 apo form dimerization), the initial state 2 (IS^PP^ apo form dimerization), the final state (5J89 holo form dimerization) respectively, T_1_ and T_2_ denote the three-body separation transition state and the “BMS-202 inserting process” transition state respectively. The relative free energy of the monomer in each state used in GITR theory is equal to the corresponding $\Delta G_{bind}$ of state divided by the monomer number (according to Eq. 26), marked in black in Figure 7.

According to GITR theory proposed by us, under certain approximate conditions, there are unified path rate relations in the following form:

$$R_{st_{i}}=-k_{t_{i}s}e^{-\frac{G_{t_{i}}-G_{s}}{RT}}\times C_{s}+\sum_{s^{\prime }}L_{f_{st_{i}}}k_{t_{i}s^{\prime }}e^{-\frac{G_{t_{i}}-G_{s^{\prime }}}{RT}}\frac{\nu_{s^{\prime }}}{\nu_{s}}C_{s^{\prime }}$$ (41)

Then, the rates of the three transformation paths which are involved in the adjustment of PD-L1s from apo form dimerization (S_1_, S_2_) to holo form dimerization (S_3_) by BMS-202 are as follows

$$R_{1t_{1}}=-k_{t_{1}1}e^{-\frac{G_{t_{1}}-G_{1}}{RT}}\times C_{1}+L_{f_{1t_{1}}}\;k_{t_{1}1}e^{-\frac{G_{t_{1}}-G_{1}}{RT}}\frac{\nu_{1}}{\nu_{1}}C_{1}+L_{f_{1t_{1}}}\;k_{t_{1}2}e^{-\frac{G_{t_{1}}-G_{2}}{RT}}\frac{\nu_{2}}{\nu_{1}}C_{2}+L_{f_{1t_{1}}}\;k_{t_{1}3}e^{-\frac{G_{t_{1}}-G_{3}}{RT}}\frac{\nu_{3}}{\nu_{1}}C_{3}$$ (42)

$$R_{2t_{1}}=-k_{t_{1}2}e^{-\frac{G_{t_{1}}-G_{2}}{RT}}\times C_{2}+L_{f_{2t_{1}}}\;k_{t_{1}1}e^{-\frac{G_{t_{1}}-G_{1}}{RT}}\frac{\nu_{1}}{\nu_{2}}C_{1}+L_{f_{2t_{1}}}\;k_{t_{1}2}e^{-\frac{G_{t_{1}}-G_{2}}{RT}}\frac{\nu_{2}}{\nu_{2}}C_{2}+L_{f_{2t_{1}}}\;k_{t_{1}3}e^{-\frac{G_{t_{1}}-G_{3}}{RT}}\frac{\nu_{3}}{\nu_{2}}C_{3}$$ (43)

$$R_{2t_{2}}=-k_{t_{2}2}e^{-\frac{G_{t_{2}}-G_{2}}{RT}}\times C_{2}+L_{f_{2t_{2}}}\;k_{t_{2}2}e^{-\frac{G_{t_{2}}-G_{2}}{RT}}\frac{\nu_{2}}{\nu_{2}}C_{2}+L_{f_{2t_{2}}}k_{t_{2}3}e^{-\frac{G_{t_{2}}-G_{3}}{RT}}\frac{\nu_{3}}{\nu_{2}}C_{3}$$ (44)

where, for this system, the linking fraction matrix is $L_{f}={\left[\begin{array}{cc} k_{t_{1}1}/\sum_{s^{\prime }=1}^{3}k_{t_{1}s^{\prime }} & 0\\ k_{t_{1}2}/\sum_{s^{\prime }=1}^{3}k_{t_{1}s^{\prime }} & k_{t_{2}2}/\sum_{s^{\prime }=2}^{3}k_{t_{2}s^{\prime }}\\ k_{t_{1}3}/\sum_{s^{\prime }=1}^{3}k_{t_{1}s^{\prime }} & k_{t_{2}3}/\sum_{s^{\prime }=2}^{3}k_{t_{2}s^{\prime }} \end{array}\right]}^{n_{s}\times n_{t}}$ , and $n_{s}=3$ , $n_{t}=2$ .

For simplicity, we can further approximate that all the frequency factors $A_{t_{i}s}$ are equal, hence all the $k_{t_{i}s}=\frac{k_{B}T}{h}A_{t_{i}s}$ are equal.

Then the linking fraction matrix of this system can be simplified as $\;L_{f}={\left[\begin{array}{cc} 1/3 & 0\\ 1/3 & 1/2\\ 1/3 & 1/2 \end{array}\right]}^{n_{s}\times n_{t}}$ , i.e., $L_{f_{1t_{1}}}=1/3$ , $L_{f_{2t_{1}}}=1/3$ , $L_{f_{2t_{2}}}=1/2$ .

For this PD-L1s dimerization system, $\nu_{1}=$ 2, $\nu_{2}=$ 2, $\nu_{3}=$ 2.

Before the BMS-202 molecules are added, $C_{3}=0$ , the concentrations of other states in the system should be the equilibrium distribution, hence $C_{1}$ and $C_{2}$ can be calculated by using the monomer distribution probability $P_{s}$ in GITR, where $C_{0}$ is the analytical concentration (total concentration) of PD-L1 monomer in the system:

$$C_{1}=\frac{1}{\nu_{1}}P_{1}C_{0}=\frac{1}{\nu_{1}}\frac{e^{-\frac{G_{1}}{RT}}}{e^{-\frac{G_{1}}{RT}}+e^{-\frac{G_{2}}{RT}}}C_{0}$$ (45)

$$C_{2}=\frac{1}{\nu_{2}}P_{2}C_{0}=\frac{1}{\nu_{2}}\frac{e^{-\frac{G_{2}}{RT}}}{e^{-\frac{G_{1}}{RT}}+e^{-\frac{G_{2}}{RT}}}C_{0}$$ (46)

Then the rates of the three transformation paths above can be simplified as follows:

$$R_{1t_{1}}=-k\;\frac{1}{Z}\frac{1}{\nu_{1}}e^{-\frac{G_{t_{1}}}{RT}}\times C_{0}+\frac{1}{3}k\;\frac{1}{Z}\frac{1}{\nu_{1}}e^{-\frac{G_{t_{1}}}{RT}}\times C_{0}+\;\frac{1}{3}k\;\frac{1}{Z}\frac{1}{\nu_{1}}e^{-\frac{G_{t_{1}}}{RT}}\times C_{0}=-\frac{1}{3}k\;\frac{1}{Z}\frac{1}{\nu_{1}}e^{-\frac{G_{t_{1}}}{RT}}\times C_{0}$$ (47)

$$R_{2t_{1}}=-k\;\frac{1}{Z}\frac{1}{\nu_{2}}e^{-\frac{G_{t_{1}}}{RT}}\times C_{0}+\frac{1}{3}k\;\frac{1}{Z}\frac{1}{\nu_{2}}e^{-\frac{G_{t_{1}}}{RT}}\times C_{0}+\frac{1}{3}k\;\frac{1}{Z}\frac{1}{\nu_{2}}e^{-G_{t_{1}}/RT}\times C_{0}=-\frac{1}{3}k\;\frac{1}{Z}\frac{1}{\nu_{2}}e^{-\frac{G_{t_{1}}}{RT}}\times C_{0}$$ (48)

$$R_{2t_{2}}=-k\;\frac{1}{Z}\frac{1}{\nu_{2}}e^{-\frac{G_{t_{2}}}{RT}}\times C_{0}+\frac{1}{2}k\;\frac{1}{Z}\frac{1}{\nu_{2}}e^{-\frac{G_{t_{2}}}{RT}}\times C_{0}=-\frac{1}{2}k\;\frac{1}{Z}\frac{1}{\nu_{2}}e^{-\frac{G_{t_{2}}}{RT}}\times C_{0}$$ (49)

where $Z=e^{-\frac{G_{1}}{RT}}+e^{-\frac{G_{2}}{RT}}$ .

The minus sign represents a decrease in concentration, that is, a transformation to the final state S_3_.

When the values are substituted in, it can be found that the absolute value of $R_{2t_{2}}$ is the largest, that is, the corresponding path (S_2_ to T_2_ to S_3_) is the dominant path for the dimerization mode transformation from apo form (pre-drug-adding) to holo form (post-drug-adding).

## Conclusion

In this study, multi-scale computational simulation results and theoretical deduction systematically reveal the multiple dimerization mode transformation in the complex self-assembly system of PD-L1s with BMS-202.

In the thermodynamical aspect: Five states (two apo form dimerization modes, one holo form dimerization mode and two intermediate states) are identified as stable in the PD-L1 system. The binding site of the 4Z18 dimerization mode is mainly in the lower part (IgC domain) of PD-L1. The symmetry mismatch (between C_2v_ and σ_v_) leads to the differences of binding free energies in 5J89 dimerization mode (between stripping mode 5J89^A^ and stripping mode 5J89^B^) and two intermediate states (between IS^R^ and IS^L^). A new stable apo form dimerization mode IS^PP^ is found, which is more stable than the apo form dimerization 4Z18. The drug BMS-202 can stabilize the IS^PP^ by a hydrophobic geometric complementary stabilizing effect which can reduce the conformational flexibility of PD-L1 interface residues and enhance the correlation of inner-chain residue motion.

In the kinetic aspect, we developed the GITR theory and then applied it to characterize the PD-L1 system. The results indicate that the BMS-202 can insert into and stabilize the apo dimerization mode IS^PP^ (initial state 2) to form the final holo dimerization mode, and the “BMS-202 inserting process” is the dominant path of inter-state transformation in the PD-L1 system. Therefore, the drug BMS-202 acts more as a stabilizer than an inducer in the process of forming the holo form dimerization mode 5J89. Moreover, we give unified mathematical expression of the transformation rate of any path and state with an integral time-dependent evolutionary analytic solution in this type of complex self-assembly network which has arbitrary finite stable states, arbitrary finite transition states, arbitrary links between them, and arbitrary kinetic orders.

Our work would contribute to a better understanding of other complex multiple-states self-assembly network systems, both as an example and at the theoretical level.

## Data Availability

The datasets presented in this study can be found in online repositories. The names of the repository/repositories and accession number(s) can be found below: https://www.rcsb.org/structure/5J89 https://www.rcsb.org/structure/4Z18.

## References

[B1] Adamcik J. Castelletto V. Bolisetty S. Hamley I. W. Mezzenga R. (2011). Direct Observation of Time-Resolved Polymorphic States in the Self-Assembly of End-Capped Heptapeptides. Angew. Chemie-International Edition 50 (24), 5495–5498. 10.1002/anie.201100807 21538748

[B2] Alder B. J. Wainwright T. E. (1959). Studies in Molecular Dynamics. I. General Method. J. Chem. Phys. 31 (2), 459–466. 10.1063/1.1730376

[B3] Andersson M. P. Uvdal P. (2005). New Scale Factors for Harmonic Vibrational Frequencies Using the B3LYP Density Functional Method with the Triple-ζ Basis Set 6-311+ G (D,p). The J. Phys. Chem. A 109 (12), 2937–2941. 16833612 10.1021/jp045733a

[B4] Baker N. A. Sept D. Joseph S. Holst M. J. McCammon J. A. (2001). Electrostatics of Nanosystems: Application to Microtubules and the Ribosome. Proc. Natl. Acad. Sci. United States America 98 (18), 10037–10041. 10.1073/pnas.181342398 PMC56910 11517324

[B5] Basu S. Yang J. Xu B. Magiera-Mularz K. Skalniak L. Musielak B. (2019). Design, Synthesis, Evaluation, and Structural Studies of C2-Symmetric Small Molecule Inhibitors of Programmed Cell Death-1/Programmed Death-Ligand 1 Protein-Protein Interaction. J. Med. Chem. 62 (15), 7250–7263. 10.1021/acs.jmedchem.9b00795 31298541

[B6] Berendsen H. J. C. Postma J. P. M. van Gunsteren W. F. DiNola A. Haak J. R. (1984). Molecular Dynamics with Coupling to an External bath. J. Chem. Phys. 81 (8), 3684–3690. 10.1063/1.448118

[B7] Brahmer J. R. Tykodi S. S. Chow L. Q. M. Hwu W.-J. Topalian S. L. Hwu P. (2012). Safety and Activity of Anti-PD-L1 Antibody in Patients with Advanced Cancer. N. Engl. J. Med. 366 (26), 2455–2465. 10.1056/nejmoa1200694 22658128 PMC3563263

[B8] Case D. A. Betz R. M. Cerutti D. S. (2016). AMBER 2016. San Francisco: University of California.

[B9] Chames P. Van Regenmortel M. Weiss E. Baty D. (2009). Therapeutic Antibodies: Successes, Limitations and Hopes for the Future. Br. J. Pharmacol. 157 (2), 220–233. 10.1111/j.1476-5381.2009.00190.x 19459844 PMC2697811

[B10] Chen Y. Liu P. Gao F. Cheng H. Qi J. Gao G. F. (2010). A Dimeric Structure of PD-L1: Functional Units or Evolutionary Relics? Protein Cell 1 (2), 153–160. 10.1007/s13238-010-0022-1 21203985 PMC4875164

[B11] Cheng L. Englander O. Paravastu A. Oates W. S. (2011). An Effective Continuum Approach for Modeling Non-equilibrium Structural Evolution of Protein Nanofiber Networks. J. Chem. Phys. 135 (5), 055102. 10.1063/1.3622489 21823733

[B12] Chupak L. S. Ding M. Martin S. W. Zheng X. Hewawasam P. Connolly T. P. (2017). Compounds Useful as Immunomodulators. U.S. Patent 9, 12–26. US 9872852.

[B13] Chupak L. S. Zheng X. (2018). Compounds Useful as Immunomodulators. U.S. Patent 9, 1–23. US 9850225.

[B14] Cooper J. A. Buhle E. L. Jr Walker S. B. Tsong T. Y. Pollard T. D. (1983). Kinetic Evidence for a Monomer Activation Step in Actin Polymerization. Biochemistry 22 (9), 2193–2202. 10.1021/bi00278a021 6860660

[B15] Dai B. Sargent C. J. Gui X. R. Liu C. Zhang F. Z. (2019). Fibril Self-Assembly of Amyloid-Spider Silk Block Polypeptides. Biomacromolecules 20 (5), 2015–2023. 10.1021/acs.biomac.9b00218 30995840

[B16] Darden T. York D. Pedersen L. (1993). Particle Mesh Ewald: an N, Log (N) Method for Ewald Sums in Large Systems. J. Chem. Phys. 98 (12), 10089–10092. 10.1063/1.464397

[B17] Eyring H. (1935). The Activated Complex and the Absolute Rate of Chemical Reactions. Chem. Rev. 17 (1), 65–77. 10.1021/cr60056a006

[B18] Fernandez-Ramos A. Miller J. A. Klippenstein S. J. Truhlar D. G. (2006). Modeling the Kinetics of Bimolecular Reactions. Chem. Rev. 106 (11), 4518–4584. 10.1021/cr050205w 17091928

[B19] Fogolari F. Zuccato P. Esposito G. Viglino P. (1999). Biomolecular Electrostatics with the Linearized Poisson-Boltzmann Equation. Biophysical J. 76 (1), 1–16. 10.1016/s0006-3495(99)77173-0 PMC1302495 9876118

[B20] Frieden C. (1985). Actin and Tubulin Polymerization: the Use of Kinetic Methods to Determine Mechanism. Annu. Rev. Biophys. biophysical Chem. 14 (1), 189–210. 10.1146/annurev.bb.14.060185.001201 3890879

[B21] Frisch M. Trucks G. Schlegel H. B. Scuseria G. E. Robb M. A. Cheeseman J. R. (2009). Gaussian 09, Revision D. 01. Wallingford CT: Gaussian. Inc., 201.

[B22] Fu J. Guerette P. A. Miserez A. (2015). Self-Assembly of Recombinant Hagfish Thread Keratins Amenable to a Strain-Induced Alpha-Helix to Beta-Sheet Transition. Biomacromolecules 16 (8), 2327–2339. 10.1021/acs.biomac.5b00552 26102237

[B23] Gordon J. C. Myers J. B. Folta T. Shoja V. Heath L. S. Onufriev A. (2005). H++: a Server for Estimating pKas and Adding Missing Hydrogens to Macromolecules. Nucleic Acids Res. 33, W368–W371. 10.1093/nar/gki464 15980491 PMC1160225

[B24] Grzybowski B. A. Fitzner K. Paczesny J. Granick S. (2017). From Dynamic Self-Assembly to Networked Chemical Systems. Chem. Soc. Rev. 46 (18), 5647–5678. 10.1039/c7cs00089h 28703815

[B25] Guzik K. Zak K. M. Grudnik P. Magiera K. Musielak B. Törner R. (2017). Small-Molecule Inhibitors of the Programmed Cell Death-1/Programmed Death-Ligand 1 (PD-1/pd-L1) Interaction via Transiently Induced Protein States and Dimerization of PD-L1. J. Med. Chem. 60 (13), 5857–5867. 10.1021/acs.jmedchem.7b00293 28613862

[B26] Hagan M. F. Chandler D. (2006). Dynamic Pathways for Viral Capsid Assembly. Biophysical J. 91 (1), 42–54. 10.1529/biophysj.105.076851 PMC1479078 16565055

[B27] Hoos A. (2016). Development of Immuno-Oncology Drugs - from CTLA4 to PD1 to the Next Generations. Nat. Rev. Drug Discov. 15 (4), 235–247. 10.1038/nrd.2015.35 26965203

[B28] Hu H. G. Li Y. M. (2020). Emerging Adjuvants for Cancer Immunotherapy. Front. Chem. 8, 601. 10.3389/fchem.2020.00601 32850636 PMC7406886

[B29] Humphrey W. Dalke A. Schulten K. (1996). VMD: Visual Molecular Dynamics. J. Mol. graphics 14 (133-8), 27–28. 10.1016/0263-7855(96)00018-5 8744570

[B30] Hunenberger P. H. Mark A. E. van Gunsteren W. F. (1995). Fluctuation and Cross-Correlation Analysis of Protein Motions Observed in Nanosecond Molecular Dynamics Simulations. J. Mol. Biol. 252 (4), 492–503. 10.1006/jmbi.1995.0514 7563068

[B31] Ishida Y. Agata Y. Shibahara K. Honjo T. (1992). Induced Expression of PD-1, a Novel Member of the Immunoglobulin Gene Superfamily, upon Programmed Cell Death. EMBO J. 11 (11), 3887–3895. 10.1002/j.1460-2075.1992.tb05481.x 1396582 PMC556898

[B32] Jakalian A. Bush B. L. Jack D. B. Bayly C. I. (2000). Fast, Efficient Generation of High-Quality Atomic Charges. AM1-BCC Model: I. Method. J. Comput. Chem. 21 (2), 132–146. 10.1002/(sici)1096-987x(20000130)21:2<132:aid-jcc5>3.0.co;2-p 12395429

[B33] Jiang L. Prentiss M. (2014). RecA-mediated Sequence Homology Recognition as an Example of How Searching Speed in Self-Assembly Systems Can Be Optimized by Balancing Entropic and Enthalpic Barriers. Phys. Rev. E 90 (2), 022704. 10.1103/PhysRevE.90.022704 PMC4972340 25215755

[B34] Jones M. R. Liu C. Wilson A. K. (2014). Molecular Dynamics Studies of the Protein-Protein Interactions in Inhibitor of Kappa B Kinase-Beta. J. Chem. Inf. Model. 54 (2), 562–572. 10.1021/ci400720n 24437505

[B35] Jorgensen W. L. Chandrasekhar J. Madura J. D. (1983). Comparison of Simple Potential Functions for Simulating Liquid Water. J. Chem. Phys. 79 (926), 926–935. 10.1063/1.445869

[B36] Kentsis A. Borden K. L. B. (2004). Physical Mechanisms and Biological Significance of Supramolecular Protein Self-Assembly. Curr. Protein Pept. Sci. 5 (2), 125–134. 10.2174/1389203043486856 15078223

[B37] Mahoney K. M. Rennert P. D. Freeman G. J. (2015). Combination Cancer Immunotherapy and New Immunomodulatory Targets. Nat. Rev. Drug Discov. 14 (8), 561–584. 10.1038/nrd4591 26228759

[B38] Maier J. A. Martinez C. Kasavajhala K. Wickstrom L. Hauser K. E. Simmerling C. (2015). ff14SB: Improving the Accuracy of Protein Side Chain and Backbone Parameters from ff99SB. J. Chem. Theor. Comput. 11 (8), 3696–3713. 10.1021/acs.jctc.5b00255 PMC4821407 26574453

[B39] Martinez-Avila O. Wu S. P. Kim S. J. Cheng Y. F. Khan F. Samudrala R. (2012). Self-Assembly of Filamentous Amelogenin Requires Calcium and Phosphate: From Dimers via Nanoribbons to Fibrils. Biomacromolecules 13 (11), 3494–3502. 10.1021/bm300942c 22974364 PMC3496023

[B40] Mccammon J. A. Gelin B. R. Karplus M. Wolynes P. G. (1976). The Hinge-Bending Mode in Lysozyme. Nature 262 (5566), 325–326. 10.1038/262325a0 958384

[B41] Michaels T. C. T. Saric A. Habchi J. Chia S. Meisl G. Vendruscolo M. (2018). Chemical Kinetics for Bridging Molecular Mechanisms and Macroscopic Measurements of Amyloid Fibril Formation. Annu. Rev. Phys. Chem. 6969, 273–298. 10.1146/annurev-physchem-050317-021322 29490200

[B42] Miller B. R. McGee T. D. Swails J. M. Homeyer N. Gohlke H. Roitberg A. E. (2012). MMPBSA.py: An Efficient Program for End-State Free Energy Calculations. J. Chem. Theor. Comput. 8 (9), 3314–3321. 10.1021/ct300418h 26605738

[B43] Miller J. F. A. P. Sadelain M. (2015). The Journey from Discoveries in Fundamental Immunology to Cancer Immunotherapy. Cancer Cell 27 (4), 439–449. 10.1016/j.ccell.2015.03.007 25858803

[B44] Nayeem S. M. Oteri F. Baaden M. Deep S. (2017). Residues of Alpha Helix H3 Determine Distinctive Features of Transforming Growth Factor Beta 3. J. Phys. Chem. B 121 (22), 5483–5498. 10.1021/acs.jpcb.7b01867 28497965

[B45] Odde D. J. Cassimeris L. Buettner H. M. (1995). Kinetics of Microtubule Catastrophe Assessed by Probabilistic Analysis. Biophysical J. 69 (3), 796–802. 10.1016/s0006-3495(95)79953-2 PMC1236309 8519980

[B46] Ozer G. Quirk S. Hernandez R. (2012). Adaptive Steered Molecular Dynamics: Validation of the Selection Criterion and Benchmarking Energetics in Vacuum. J. Chem. Phys. 136 (21), 215104. 10.1063/1.4725183 22697572

[B47] Ozer G. Valeev E. F. Quirk S. Hernandez R. (2010). Adaptive Steered Molecular Dynamics of the Long-Distance Unfolding of Neuropeptide Y. J. Chem. Theor. Comput. 6 (10), 3026–3038. 10.1021/ct100320g 26616767

[B48] Pashuck E. T. Stupp S. I. (2010). Direct Observation of Morphological Tranformation from Twisted Ribbons into Helical Ribbons. J. Am. Chem. Soc. 132 (26), 8819–8821. 10.1021/ja100613w 20552966 PMC3116515

[B49] Pitre S. Alamgir M. Green J. R. Dumontier M. Dehne F. Golshani A. (2008). “Computational Methods for Predicting Protein-Protein Interactions,” in Protein - Protein Interaction. Editors Werther M. Seitz H., 110, 247–267. 10.1007/10_2007_089 18202838

[B50] Rha G. B. Wu G. Shoelson S. E. Chi Y. I. (2009). Multiple Binding Modes between HNF4 Alpha and the LXXLL Motifs of PGC-1 Alpha Lead to Full Activation. J. Biol. Chem. 284 (50), 35165–35176. 10.1074/jbc.m109.052506 19846556 PMC2787377

[B51] Roe D. R. Cheatham T. E. (2013). PTRAJ and CPPTRAJ: Software for Processing and Analysis of Molecular Dynamics Trajectory Data. J. Chem. Theor. Comput. 9 (7), 3084–3095. 10.1021/ct400341p 26583988

[B52] Ross J. Vlad M. O. (1999). Nonlinear Kinetics and New Approaches to Complex Reaction Mechanisms. Annu. Rev. Phys. Chem. 50, 51–78. 10.1146/annurev.physchem.50.1.51 15012406

[B53] Ryckaert J.-P. Ciccotti G. Berendsen H. J. (1977). Numerical Integration of the Cartesian Equations of Motion of a System with Constraints: Molecular Dynamics of N-Alkanes. J. Comput. Phys. 23, 327–341. 10.1016/0021-9991(77)90098-5

[B54] Saric A. Michaelsa T. C. T. Zaccone A. Knowles T. P. J. Frenkel D. (2016). Kinetics of Spontaneous Filament Nucleation via Oligomers: Insights from Theory and Simulation. J. Chem. Phys. 145 (21), 211926. 10.1063/1.4965040 28799382

[B55] Sharma P. Allison J. P. (2015). The Future of Immune Checkpoint Therapy. Science 348 (6230), 56–61. 10.1126/science.aaa8172 25838373

[B56] Shi D. F. An X. L. Bai Q. F. Bing Z. T. Zhou S. Y. Liu H. X. (2019). Computational Insight into the Small Molecule Intervening PD-L1 Dimerization and the Potential Structure-Activity Relationship. Front. Chem. 7. 764. 10.3389/fchem.2019.00764 31781546 PMC6861162

[B57] Sitkoff D. Sharp K. A. Honig B. (1994). Accurate Calculation of Hydration Free Energies Using Macroscopic Solvent Models. J. Phys. Chem. 98 (7), 1978–1988. 10.1021/j100058a043

[B58] Skalniak L. Zak K. M. Guzik K. Magiera K. Musielak B. Pachota M. (2017). Small-molecule Inhibitors of PD-1/pd-L1 Immune Checkpoint Alleviate the PD-L1-Induced Exhaustion of T-Cells. Oncotarget 8 (42), 72167–72181. 10.18632/oncotarget.20050 29069777 PMC5641120

[B59] Soremekun O. S. Olotu F. A. Agoni C. Soliman M. E. S. (2019). Recruiting Monomer for Dimer Formation: Resolving the Antagonistic Mechanisms of Novel Immune Check point Inhibitors against Programmed Death Ligand-1 in Cancer Immunotherapy. Mol. Simulation 45 (10), 777–789. 10.1080/08927022.2019.1593977

[B60] Sun X. Liang L. Gu J. K. Zhuo W. Yan X. Xie T. (2019). Inhibition of Programmed Cell Death Protein Ligand-1 (PD-L1) by Benzyl Ether Derivatives: Analyses of Conformational Change, Molecular Recognition and Binding Free Energy. J. Biomol. Struct. Dyn. 37 (18), 4801–4812. 10.1080/07391102.2018.1563568 30593257

[B61] Topalian S. L. Hodi F. S. Brahmer J. R. Gettinger S. N. Smith D. C. McDermott D. F. (2012). Safety, Activity, and Immune Correlates of Anti-PD-1 Antibody in Cancer. N. Engl. J. Med. 366 (26), 2443–2454. 10.1056/nejmoa1200690 22658127 PMC3544539

[B62] Tuckerman M. E. Martyna G. J. (2000). Understanding Modern Molecular Dynamics: Techniques and Applications. J. Phys. Chem. B 104 (2), 159–178. 10.1021/jp992433y

[B63] Uberuaga B. P. Anghel M. Voter A. F. (2004). Synchronization of Trajectories in Canonical Molecular-Dynamics Simulations: Observation, Explanation, and Exploitation. J. Chem. Phys. 120 (14), 6363–6374. 10.1063/1.1667473 15267525

[B64] Wang J. Wolf R. M. Caldwell J. W. Kollman P. A. Case D. A. (2004). Development and Testing of a General Amber Force Field. J. Comput. Chem. 25 (9), 1157–1174. 10.1002/jcc.20035 15116359

[B65] Zak K. M. Grudnik P. Guzik K. Zieba B. J. Musielak B. Dömling A. (2016). Structural Basis for Small Molecule Targeting of the Programmed Death Ligand 1 (PD-L1). Oncotarget 7 (21), 30323–30335. 10.18632/oncotarget.8730 27083005 PMC5058683

[B66] Zhang F. Wei H. Wang X. Bai Y. Wang P. Wu J. (2017). Structural Basis of a Novel PD-L1 Nanobody for Immune Checkpoint Blockade. Cell Discov 3, 17004. 10.1038/celldisc.2017.4 28280600 PMC5341541

[B67] Zierenberg J. Schierz P. Janke W. (2017). Canonical Free-Energy Barrier of Particle and Polymer Cluster Formation. Nat. Commun. 8 (1), 1–7. 10.1038/ncomms14546 28240262 PMC5333364

