# Supplementary material for: Predicting a Kind of Unusual Multiple-States Dimerization-Modes Transformation in Protein PD-L1 System by Computational Investigation and a Generalized Rate Theory
Source: Front Chem. 2021 Nov 9;9:783444. doi: 10.3389/fchem.2021.783444 (PMC8631179; doi:10.3389/fchem.2021.783444)
Supplement: Supplementary file 1 [file DataSheet1.DOCX]

Supplementary Material

Predicting a Kind of Unusual Multiple-States Dimerization-Modes Transformation in Protein PD-L1 System by Computational Investigation and a Generalized Rate Theory

#
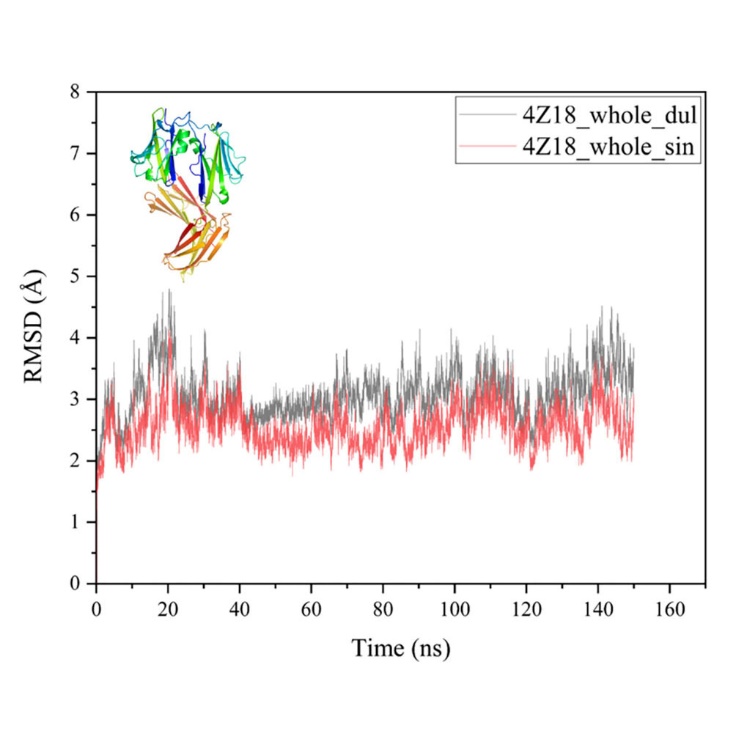


**Supplementary Figure S1.** Structural stability of the 4Z18 whole system measured by the root mean square deviation (RMSD) of all Ca atoms referencing double chains (black) and single chain (red). The structure of 4Z18 dimerization mode is shown at the top-left corner for the purpose of clarity.


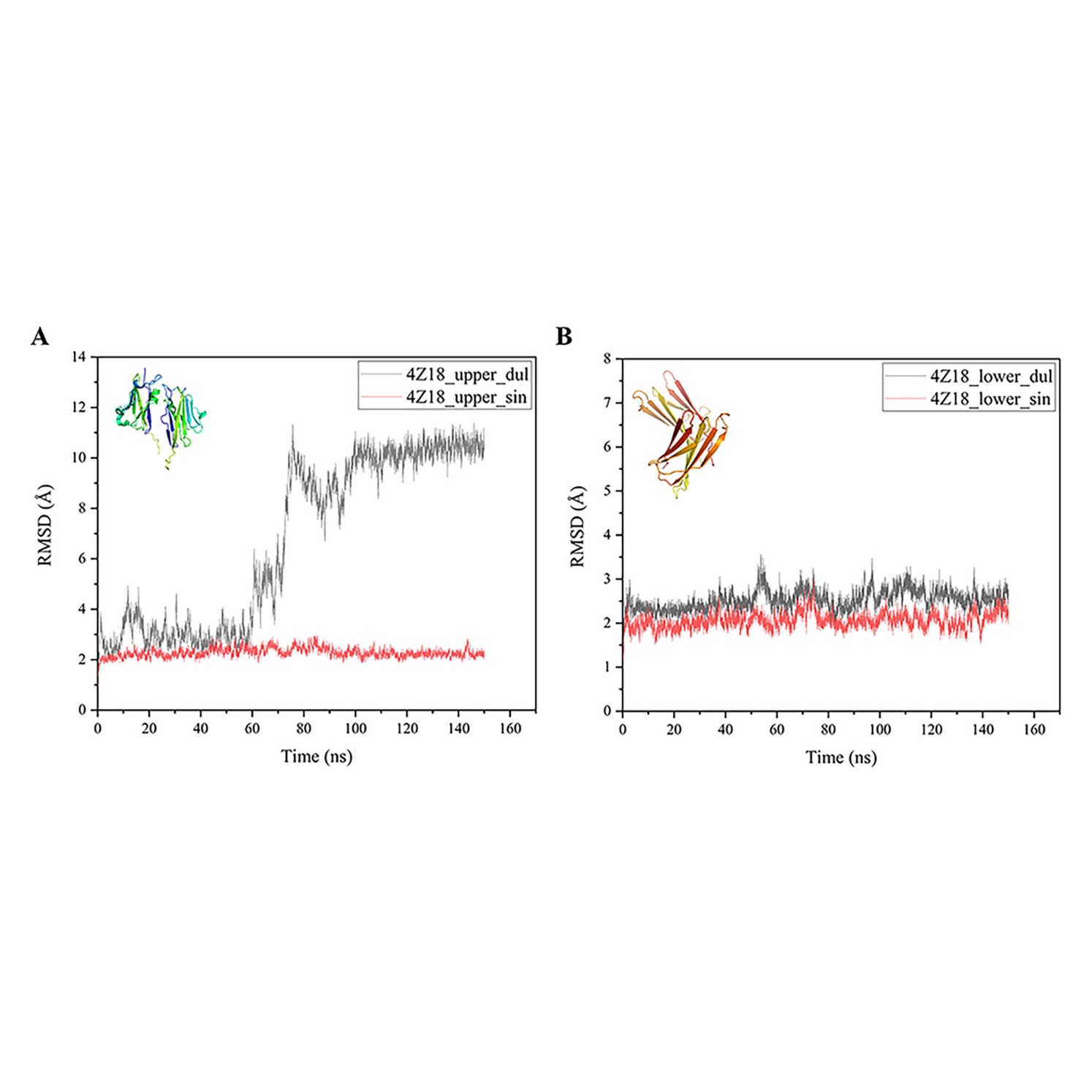


**Supplementary Figure S2.** Structural stability of the 4Z18 upper system (A) and lower system (B) measured by the root mean square deviation (RMSD) of all Ca atoms referencing double chains (black) and single chain (red). The structures of dimerization modes of 4Z18 upper system and lower system are shown at the top-left corner respectively for the purpose of clarity.


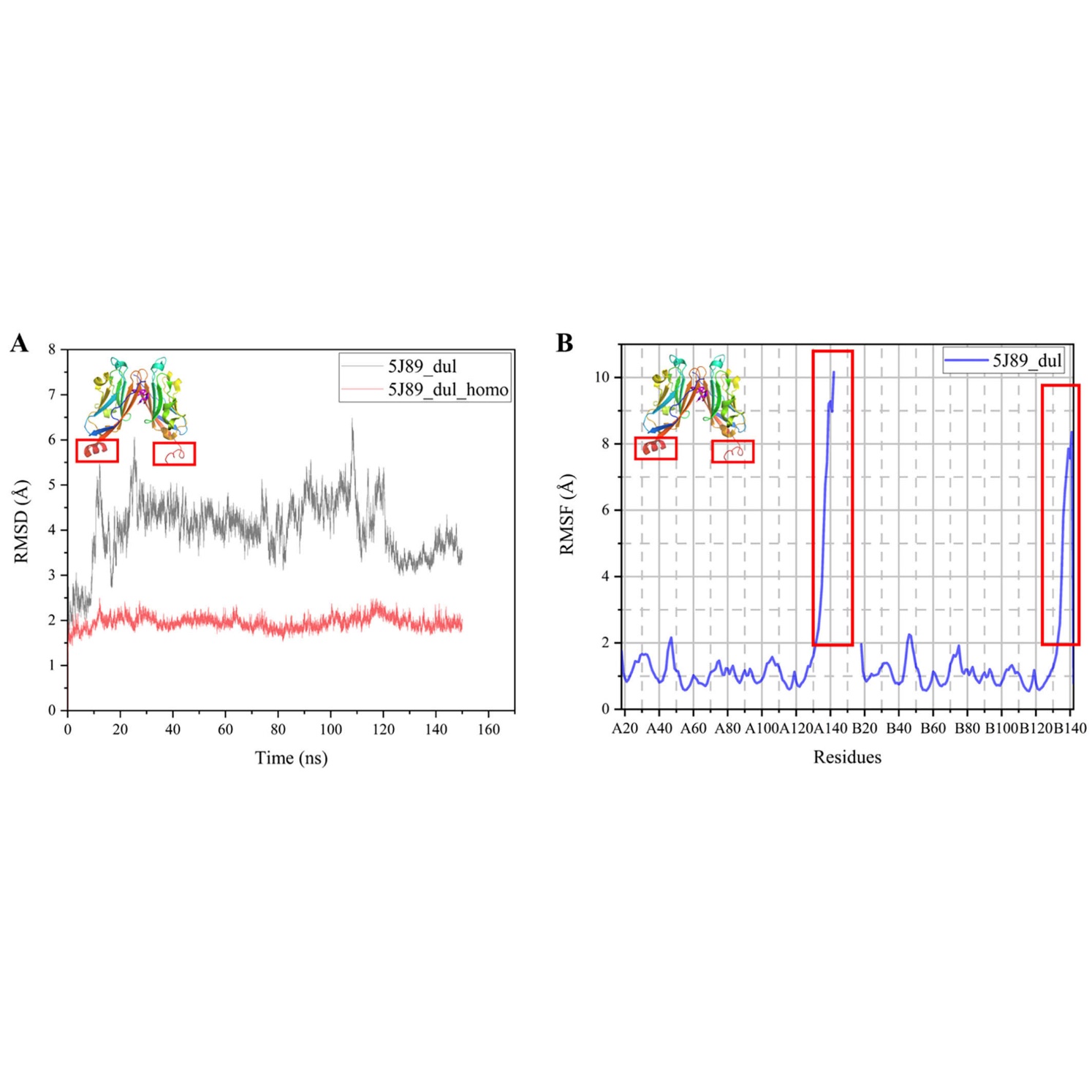


**Supplementary Figure S3.** Structural stability (A) in the 5J89 system and residue fluctuations (B). The root mean square deviation (RMSD) of all Ca atoms referencing double chains (black) and single chain (red). The root mean square fluctuations (RMSF) of residues (blue). The structure at the top-left corner shows the 5J89 is a kind of sandwich structure, with the small-molecular drug BMS-202 (purple stick model) entrapped between two PD-L1 monomers. The red boxes are 10-residue-long disordered structure at the end of two chains with high fluctuation.


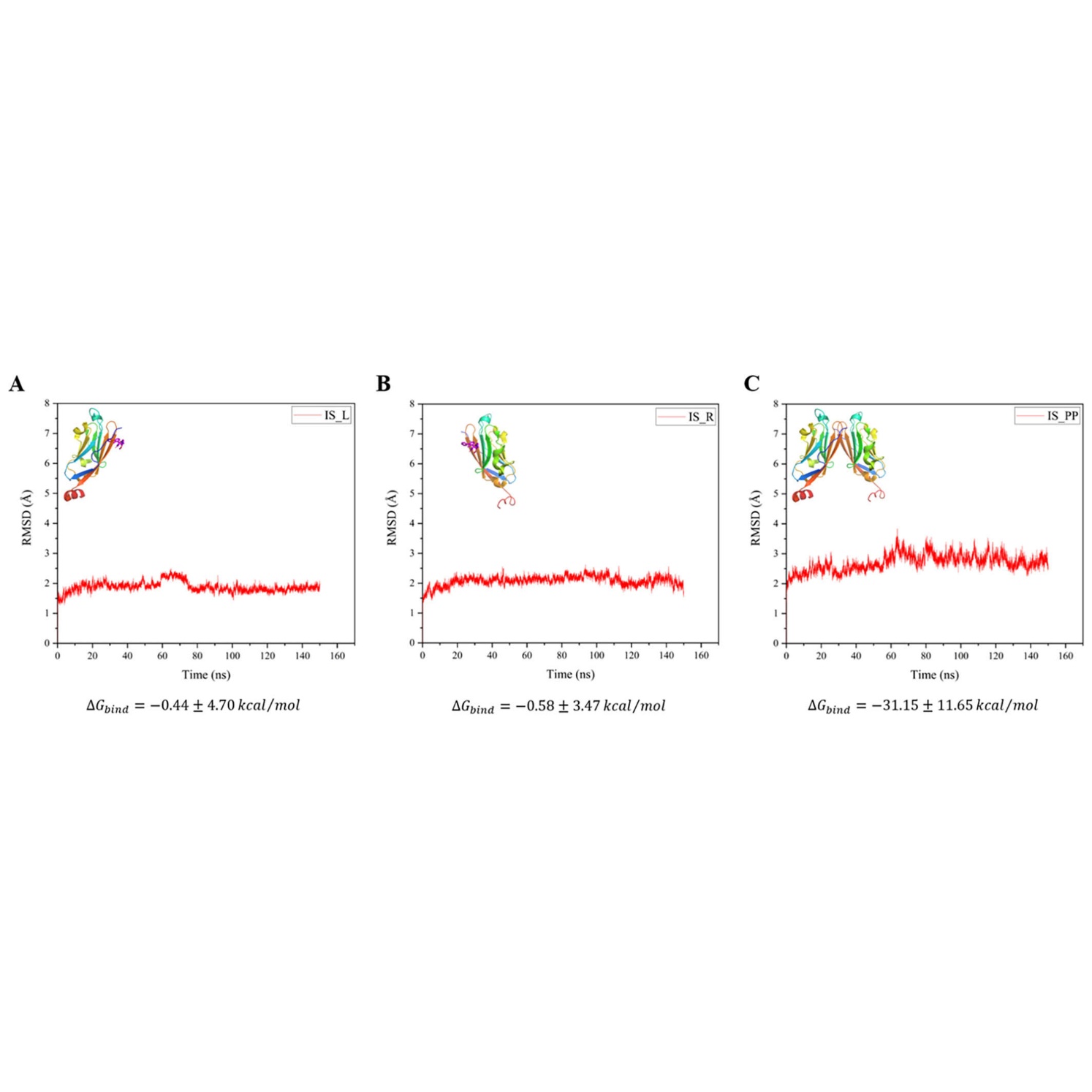


**Supplementary Figure S4.** The RMSD and the binding free energies of IS^L^ (A), IS^R^ (B) and IS^PP^ (C).


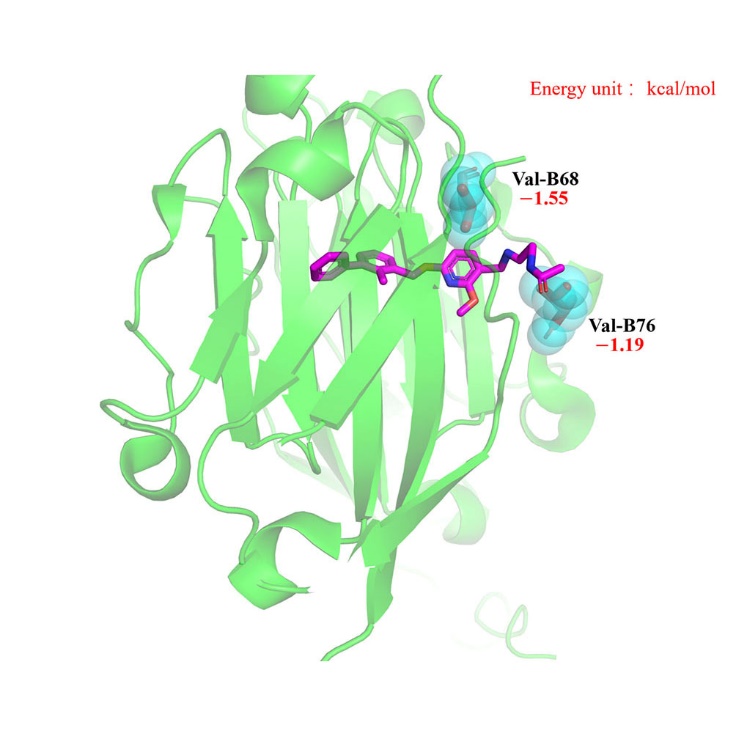


**Supplementary Figure S5.** The structure and free energies contribution of Val-B68 and Val-B76 in intermediate IS^R^.


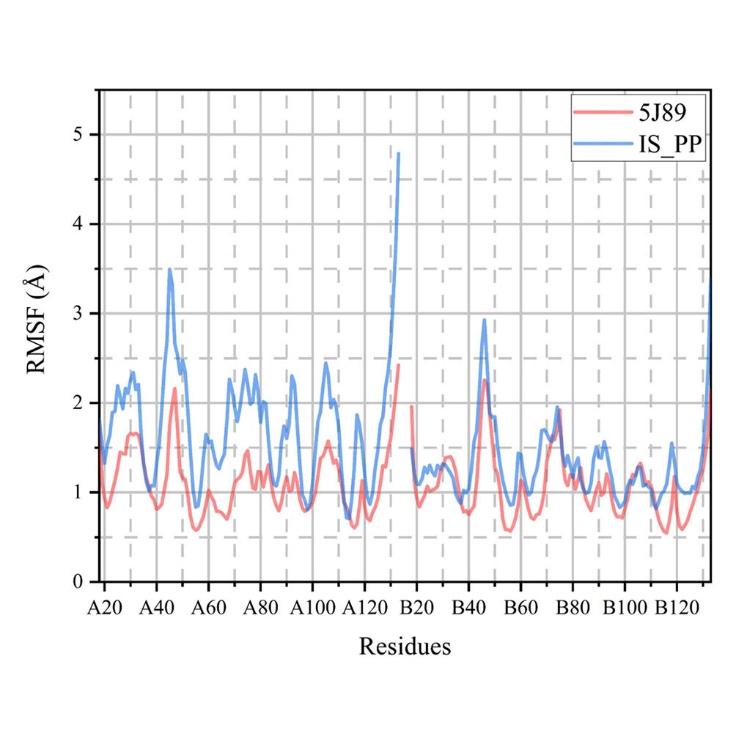


**Supplementary Figure S6.** The root mean square fluctuations (RMSF) of homo residues in 5J89 system (red) and IS^PP^ system (blue).


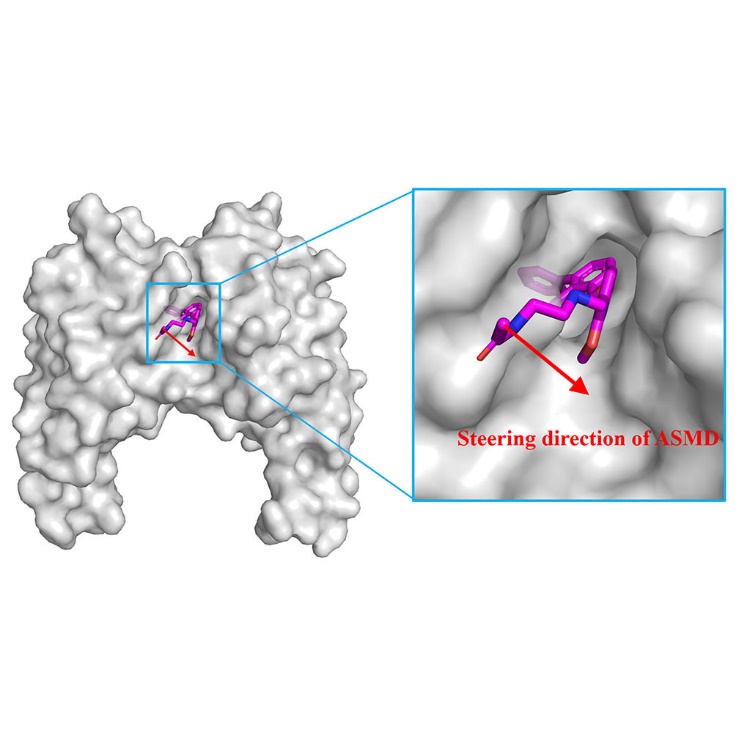


**Supplementary Figure S7.** The cavity between the binding interface of two PD-L1s. The BMS-202 structure is shown as stick model (purple). The steering direction (red arrow) of ASMD.


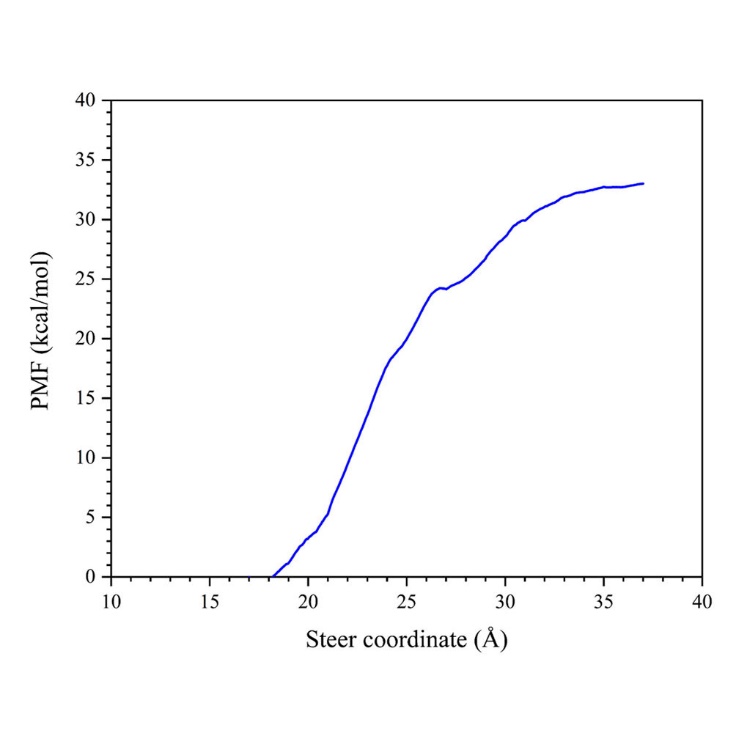


**Supplementary Figure S8.** The potential of mean force (PMF) of ASMD in 5J89 system.


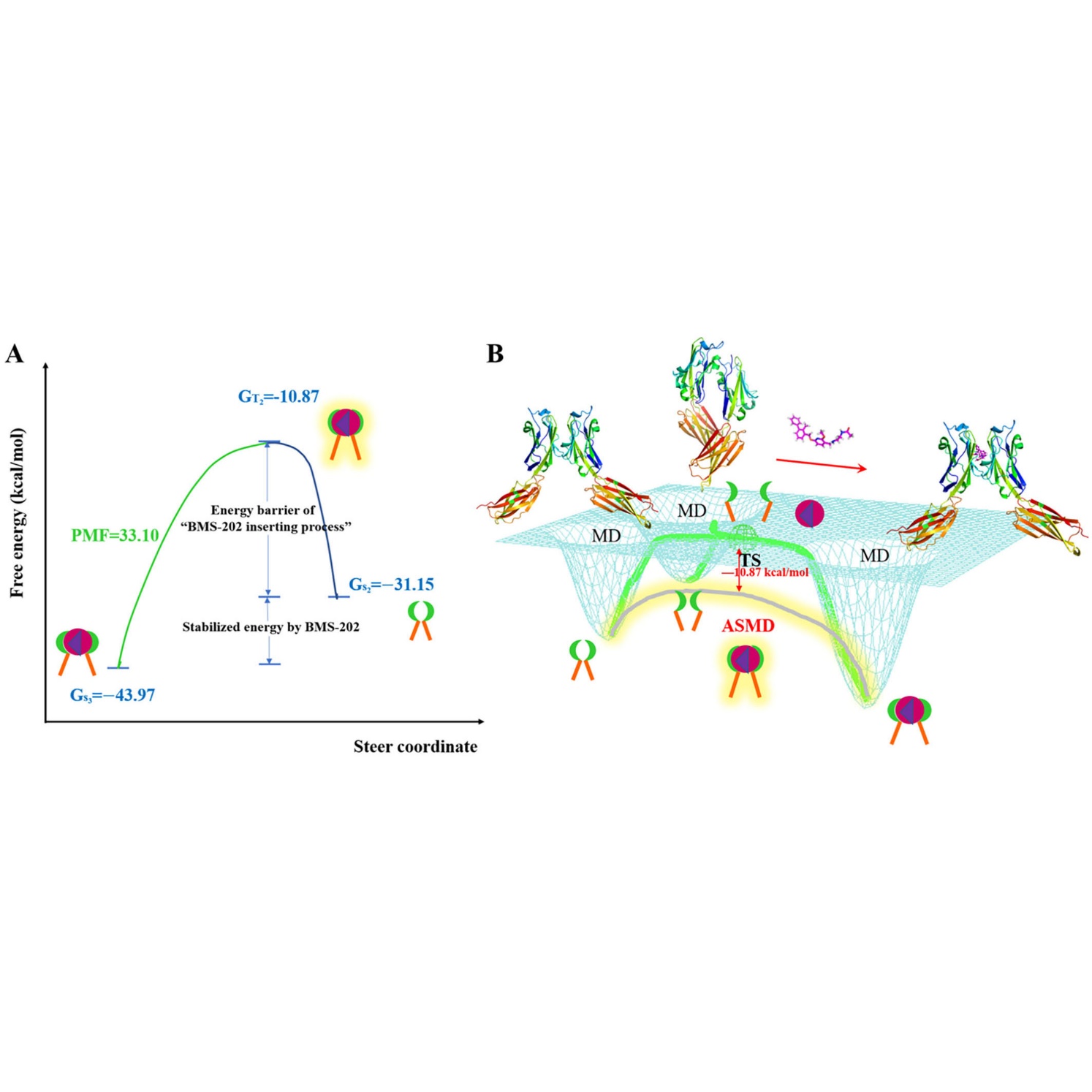


**Supplementary Figure S9.** (A) The relation between the PMF of the pull-out process and the free energy surface profile of the “BMS-202 inserting process”. (B) The integral free energy surface and the general view of transition paths between stable dimerization modes in PD-L1s system. The green crescent and the orange bar denote the upper domain and the lower domain of PD-L1 respectively. The purple disk denotes the drug BMS-202 with the triangle breaking integral symmetry.

**Supplementary Table S1.** The binding free energies (kcal mol^-1^) and its components for the dimerization 4Z18.

|  | 4Z18 |
| --- | --- |
| ΔE_vdw_ | $-$113.96$\pm$6.04 |
| ΔE_ele_ | $-$456.16$\pm$38.18 |
| ΔG_PB_ | 487.86$\pm$36.40 |
| ΔG_SA_ | $-$14.65$\pm$0.42 |
| ΔE_polar_ ^a^ | 31.70$\pm$52.75 |
| ΔE_nonpolar_ ^b^ | $-$128.61$\pm$6.05 |
| ΔG_mmpbsa_ ^c^ | $-$96.91$\pm$9.58 |
| TΔ**S** | $-$73.28$\pm$4.24 |
| **Δ**G_bind_ | $-$23.63$\pm$10.48 |

**^a^** ΔE_polar_=ΔE_ele_+ΔG_PB_

**^b^** ΔE_nonpolar_=ΔE_vdw_+ΔG_SA_

**^c^** ΔG_mmpbsa_=ΔE_vdw_+ΔE_ele_+ΔG_PB_+ΔG_SA_=ΔE_polar_+ΔE_nonpolar_

**Supplementary Table S2.** The binding free energies (kcal mol^-1^) and its components for the dimerization 4Z18 upper system.

|  | 4Z18 upper system |
| --- | --- |
| ΔE_vdw_ | $-$36.73$\pm$8.47 |
| ΔE_ele_ | $-$100.23$\pm$35.51 |
| ΔG_PB_ | 123.30$\pm$35.39 |
| ΔG_SA_ | $-$4.29$\pm$1.04 |
| ΔE_polar_ ^a^ | 23.07$\pm$50.13 |
| ΔE_nonpolar_ ^b^ | $-$41.02$\pm$8.53 |
| ΔG_mmpbsa_ ^c^ | $-$17.94$\pm$8.54 |
| TΔ**S** | $-$40.65$\pm$0.32 |
| **Δ**G_bind_ | 22.71±8.86 |

**^a^** ΔE_polar_=ΔE_ele_+ΔG_PB_

**^b^** ΔE_nonpolar_=ΔE_vdw_+ΔG_SA_

**^c^** ΔG_mmpbsa_=ΔE_vdw_+ΔE_ele_+ΔG_PB_+ΔG_SA_=ΔE_polar_+ΔE_nonpolar_

**Supplementary Table S3.** The binding free energies (kcal mol^-1^) and its components for the dimerization 4Z18 lower system.

|  | 4Z18 lower system |
| --- | --- |
| ΔE_vdw_ | $-$63.78$\pm$5.57 |
| ΔE_ele_ | $-$392.79$\pm$69.99 |
| ΔG_PB_ | 400.94$\pm$65.83 |
| ΔG_SA_ | $-$7.57$\pm$0.55 |
| ΔE_polar_ ^a^ | 8.15$\pm$96.08 |
| ΔE_nonpolar_ ^b^ | $-$71.35$\pm$5.60 |
| ΔG_mmpbsa_ ^c^ | $-$63.20$\pm$9.38 |
| TΔ**S** | $-$41.58$\pm$0.28 |
| **Δ**G_bind_ | $-$21.62±9.66 |

**^a^** ΔE_polar_=ΔE_ele_+ΔG_PB_

**^b^** ΔE_nonpolar_=ΔE_vdw_+ΔG_SA_

**^c^** ΔG_mmpbsa_=ΔE_vdw_+ΔE_ele_+ΔG_PB_+ΔG_SA_=ΔE_polar_+ΔE_nonpolar_

**Supplementary Table S4.** The binding free energies (kcal mol^-1^) and its components for the 5J89 system with the stripping mode A.

|  | 5J89^A^ |
| --- | --- |
| ΔE_vdw_ | $-$73.59$\pm$6.59 |
| ΔE_ele_ | $-$301.55$\pm$48.10 |
| ΔG_PB_ | 300.79$\pm$40.91 |
| ΔG_SA_ | $-$7.67$\pm$0.56 |
| ΔE_polar_ ^a^ | $-$0.76$\pm$63.14 |
| ΔE_nonpolar_ ^b^ | $-$81.26$\pm$6.61 |
| ΔG_mmpbsa_ ^c^ | $-$82.02$\pm$13.91 |
| TΔ**S** | $-$43.75$\pm$3.43 |
| **Δ**G_bind_ | $-$38.27±14.33 |

**^a^** ΔE_polar_=ΔE_ele_+ΔG_PB_

**^b^** ΔE_nonpolar_=ΔE_vdw_+ΔG_SA_

**^c^** ΔG_mmpbsa_=ΔE_vdw_+ΔE_ele_+ΔG_PB_+ΔG_SA_=ΔE_polar_+ΔE_nonpolar_

**Supplementary Table S5.** The binding free energies (kcal mol^-1^) and its components for the 5J89 system with the stripping mode B.

|  | 5J89^B^ |
| --- | --- |
| ΔE_vdw_ | $-$84.92$\pm$5.46 |
| ΔE_ele_ | $-$283.87$\pm$26.89 |
| ΔG_PB_ | 284.90$\pm$22.59 |
| ΔG_SA_ | $-$8.45$\pm$0.36 |
| ΔE_polar_ ^a^ | 1.03$\pm$35.12 |
| ΔE_nonpolar_ ^b^ | $-$93.37$\pm$5.47 |
| ΔG_mmpbsa_ ^c^ | $-$92.33$\pm$9.36 |
| TΔ**S** | $-$41.51$\pm$3.21 |
| **Δ**G_bind_ | $-$50.82±9.90 |

**^a^** ΔE_polar_=ΔE_ele_+ΔG_PB_

**^b^** ΔE_nonpolar_=ΔE_vdw_+ΔG_SA_

**^c^** ΔG_mmpbsa_=ΔE_vdw_+ΔE_ele_+ΔG_PB_+ΔG_SA_=ΔE_polar_+ΔE_nonpolar_

**Supplementary Table S6.** The binding free energies (kcal mol^-1^) and its components for the 5J89 system with the stripping mode C.

|  | 5J89^C^ |
| --- | --- |
| ΔE_vdw_ | $-$64.96$\pm$3.58 |
| ΔE_ele_ | $-$11.21$\pm$5.56 |
| ΔG_PB_ | 38.05$\pm$5.78 |
| ΔG_SA_ | $-$4.50$\pm$0.19 |
| ΔE_polar_ ^a^ | 26.84$\pm$8.02 |
| ΔE_nonpolar_ ^b^ | $-$69.46$\pm$3.59 |
| ΔG_mmpbsa_ ^c^ | $-$42.62$\pm$3.71 |
| TΔ**S** | $-$29.60$\pm$0.86 |
| **Δ**G_bind_ | $-$13.02±3.81 |

**^a^** ΔE_polar_=ΔE_ele_+ΔG_PB_

**^b^** ΔE_nonpolar_=ΔE_vdw_+ΔG_SA_

**^c^** ΔG_mmpbsa_=ΔE_vdw_+ΔE_ele_+ΔG_PB_+ΔG_SA_=ΔE_polar_+ΔE_nonpolar_

**Supplementary Table S7.** Properties of H-bonds between chain A and chain B in 5J89 system.

| Acceptor | Donor | Occupancy of H-bonds |
| --- | --- | --- |
| Glu-A58 | Arg-B125 | 2.59 |
| Asp-A61 | Arg-B113 | 1.51 |
| Glu-B58 | Arg-A125 | 1.19 |
| Glu-B58 | Tyr-A123 | 1.12 |
| Glu-A58 | Tyr-B123 | 1.00 |
| Asp-B122 | Tyr-A56 | 0.86 |
| Asp-A61 | Arg-B125 | 0.77 |
| Asp-B61 | Arg-A125 | 0.65 |
| Asp-B61 | Arg-A113 | 0.47 |
| Glu-B58 | Arg-A113 | 0.35 |
| Ser-B117 | Ser-A100 | 0.27 |
| Gly-B119 | His-A67 | 0.26 |
| Gly-A119 | His-B69 | 0.16 |
| Tyr-B123 | Arg-A113 | 0.12 |
| Arg-A113 | Arg-B113 | 0.06 |
| Tyr-A123 | Arg-B113 | 0.04 |
| Ala-B121 | Tyr-A56 | 0.03 |
| Tyr-A123 | Tyr-B123 | 0.02 |
| SUM |  | 11.47 |

**Supplementary Table S8.** The binding free energies (kcal mol^-1^) and its components for the IS^L^ system.

|  | IS^L^ |
| --- | --- |
| ΔE_vdw_ | $-$27.72$\pm3$.31 |
| ΔE_ele_ | $-12$.55$\pm9$.50 |
| ΔG_PB_ | 22.37$\pm$8.37 |
| ΔG_SA_ | $-$2.23$\pm$0.30 |
| ΔE_polar_ ^a^ | $9.82\pm$12.66 |
| ΔE_nonpolar_ ^b^ | $-$29.95$\pm$3.32 |
| ΔG_mmpbsa_ ^c^ | $-$20.13$\pm$4.09 |
| TΔ**S** | $-$19.69$\pm$2.31 |
| **Δ**G_bind_ | $-$0.44$\pm$4.70 |

**^a^** ΔE_polar_=ΔE_ele_+ΔG_PB_

**^b^** ΔE_nonpolar_=ΔE_vdw_+ΔG_SA_

**^c^** ΔG_mmpbsa_=ΔE_vdw_+ΔE_ele_+ΔG_PB_+ΔG_SA_=ΔE_polar_+ΔE_nonpolar_

**Supplementary Table S9.** The binding free energies (kcal mol^-1^) and its components for the IS^R^ system.

|  | IS^R^ |
| --- | --- |
| ΔE_vdw_ | $-$32.73$\pm$2.63 |
| ΔE_ele_ | $-$2.73$\pm$2.69 |
| ΔG_PB_ | 17.88$\pm$3.08 |
| ΔG_SA_ | $-$2.70$\pm$0.24 |
| ΔE_polar_ ^a^ | 15.15$\pm$4.09 |
| ΔE_nonpolar_ ^b^ | $-$35.43$\pm$2.64 |
| ΔG_mmpbsa_ ^c^ | $-$20.29$\pm$2.64 |
| TΔ**S** | $-$19.71$\pm$2.25 |
| **Δ**G_bind_ | $-$0.58$\pm$3.47 |

**^a^** ΔE_polar_=ΔE_ele_+ΔG_PB_

**^b^** ΔE_nonpolar_=ΔE_vdw_+ΔG_SA_

**^c^** ΔG_mmpbsa_=ΔE_vdw_+ΔE_ele_+ΔG_PB_+ΔG_SA_=ΔE_polar_+ΔE_nonpolar_

**Supplementary Table S10.** The binding free energies (kcal mol^-1^) and its components for the dimerization IS^PP^ system.

|  | IS^PP^ |
| --- | --- |
| ΔE_vdw_ | $-$46.19$\pm$6.19 |
| ΔE_ele_ | $-$280.86$\pm$50.21 |
| ΔG_PB_ | 264.66$\pm$42.01 |
| ΔG_SA_ | $-$6.00$\pm$0.39 |
| ΔE_polar_ ^a^ | $-$16.20$\pm$65.46 |
| ΔE_nonpolar_ ^b^ | $-$52.19$\pm$6.20 |
| ΔG_mmpbsa_ ^c^ | $-$68.40$\pm$11.22 |
| TΔ**S** | $-$37.25$\pm$3.12 |
| **Δ**G_bind_ | $-$31.15$\pm$11.65 |

**^a^** ΔE_polar_=ΔE_ele_+ΔG_PB_

**^b^** ΔE_nonpolar_=ΔE_vdw_+ΔG_SA_

**^c^** ΔG_mmpbsa_=ΔE_vdw_+ΔE_ele_+ΔG_PB_+ΔG_SA_=ΔE_polar_+ΔE_nonpolar_
